# Multiple cropping systems of the world and the potential for increasing cropping intensity

**DOI:** 10.1016/j.gloenvcha.2020.102131

**Published:** 2020-09

**Authors:** Katharina Waha, Jan Philipp Dietrich, Felix T. Portmann, Stefan Siebert, Philip K. Thornton, Alberte Bondeau, Mario Herrero

**Affiliations:** a CSIRO, Agriculture & Food, 306 Carmody Rd, St Lucia, QLD, Australia; b Potsdam Institute for Climate Impact Research, Telegrafenberg A31, 14473 Potsdam, Germany; c Goethe University Frankfurt, Institute of Physical Geography, 60438 Frankfurt am Main, Germany; d University of Göttingen, Department of Crop Sciences, Von-Siebold-Strasse 8, 37075 Göttingen, Germany; e CGIAR Research Program on Climate Change, Agriculture and Food Security (CCAFS), ILRI, PO Box 30709, Nairobi 00100, Kenya; f International Livestock Research Institute (ILRI), Nairobi 00100, Kenya; g Institut Mediterraneen de Biodiversite et d’Ecologie Marine et Continentale (IMBE), Aix-Marseille Universite, CNRS, IRD, Avignon Universite, France; h University of Göttingen, Centre of Biodiversity and Sustainable Land Use, Büsgenweg 1, 37077 Göttingen, Germany

**Keywords:** Double cropping, Land sparing, Cropping intensity, Harvest frequency, Land use intensity, Global crop production

## Abstract

Multiple cropping, defined as harvesting more than once a year, is a widespread land management strategy in tropical and subtropical agriculture. It is a way of intensifying agricultural production and diversifying the crop mix for economic and environmental benefits. Here we present the first global gridded data set of multiple cropping systems and quantify the physical area of more than 200 systems, the global multiple cropping area and the potential for increasing cropping intensity. We use national and sub-national data on monthly crop-specific growing areas around the year 2000 (1998–2002) for 26 crop groups, global cropland extent and crop harvested areas to identify sequential cropping systems of two or three crops with non-overlapping growing seasons. We find multiple cropping systems on 135 million hectares (12% of global cropland) with 85 million hectares in irrigated agriculture. 34%, 13% and 10% of the rice, wheat and maize area, respectively are under multiple cropping, demonstrating the importance of such cropping systems for cereal production. Harvesting currently single cropped areas a second time could increase global harvested areas by 87–395 million hectares, which is about 45% lower than previous estimates. Some scenarios of intensification indicate that it could be enough land to avoid expanding physical cropland into other land uses but attainable intensification will depend on the local context and the crop yields attainable in the second cycle and its related environmental costs.

## Highlights

• Global area of different multiple cropping systems quantified for the first time.
• Twelve percent of global cropland are used for growing two or three crops in a sequence.
• Potential for increasing harvest frequency smaller than previously estimated.

## 1 Introduction

Multiple cropping is common and a widespread land use management strategy in low-land tropical and subtropical agriculture where rainy seasons are long enough or irrigation is viable. Changes in cropping intensity, not just in harvested area and crop yields are associated with crop production (Cohn et al., 2016, Iizumi and Ramankutty, 2014). In the past, increases in cropping intensity resulted in an extra crop harvest globally every ten year period since 1961 (Ray and Foley, 2013) and accounted for 9% of the global crop production growth and 31% of the crop production growth in sub-Sahara Africa between 1961 and 2007 (Alexandratos and Bruinsma, 2012). Besides the direct benefit for crop production by increasing the number of harvest and the amount of biomass extracted, multiple cropping can improve the functioning of agricultural systems and reduce the environmental consequences sometimes associated with crop production (Gaba et al., 2015). Intensification occurs in systems with multiple harvests of the same crop, for example in some Asian rice-based systems, while other systems are more diverse with different types of crops grown at the same time or in a sequence and interacting with each other. It is expected that higher diversity increases the sustainability of crop production (Altieri, 1999), pest regulation (Khan et al., 1997), resistance to climate events (Isbell et al., 2015, Lin, 2011) and reduces fertilizer use in associations with legumes (Peoples et al., 2009) all of which can also lead to increases in production or profitability in the short or long term (for a review see Francis, 1986, Gaba et al., 2015). In a cropping system with diverse crop species, multiple cropping also allows for risk-spreading and diversification to different growing seasons and different crops for own use or markets. On the other hand, growing a second or third crop can increase the risk of crop failure (Ojeda et al., 2018) and the environmental costs of production (Damien et al., 2017, Ladha et al., 2003, Timsina and Connor, 2001) but these problems are crop-, location- and management-specific.

It is crucial to acknowledge that crop diversification and intensification are two different management strategies with different goals that can lead to the same level of cropping intensity, i.e. the same ratio of harvested area and physical area or the same number of harvests. We need to expand the current measures of cropping intensity by including a description of the crop mix in each system. Advancing the research in the areas outlined above using global agricultural models also requires a better representation of the spatial extent of different multiple cropping systems. This and their contribution to food production is largely unknown on the global scale. Multiple cropping systems are poorly accounted for in assessments of global food production and land use change and effects of multiple cropping on crop production, ground coverage, water fluxes, soil erosion, albedo, soil chemical properties, and pest infestation are neglected in global agricultural models. While feedbacks between different plants, crops, and micro-organisms that contribute to food production directly or indirectly are important on the field and farm scale, their influence on larger spatial scales is unknown but we expect them to be significant. Developing a crop-specific and spatially explicit multiple cropping data set and integrating into a global agricultural modelling framework will also be important to study the extent to which the suggested intensification on current cropland for increasing crop production (Ray and Foley, 2013, Wu et al., 2018) can be sustainable. Consequently the main objectives of our study are to i) develop a method to map and characterize multiple cropping systems on the macro scale from gridded crop area data sets and sub-national to national cropping calendars, ii) introduce estimates of multiple cropping area for the time period 1998–2002 as a potential baseline for future global or regional studies monitoring multiple cropping and iii) estimate the potential to increase cropping intensity.

Methodological approaches so far include remote sensing approaches, combining remote sensing data with agricultural census data and non-remote sensing approaches. They all result in useful estimates of the area under multiple cropping, or the timing of the crop cycles in different cropping systems but not both at the same time. Remote sensing approaches can identify phenological cycles, the greening up, peak period and maturity from time series of vegetation indices such as NDVI and EVI as well as crop classes and were developed for application on the global scale (Whitcraft et al., 2015, Wu et al., 2018) and for cropland in America (Arvor et al., 2011, Brown et al., 2013, Galford et al., 2008, Wardlow et al., 2007), Europe (Belgiu and Csillik, 2018, Estel et al., 2016), Africa (Xiong et al., 2017) and Asia (Gray et al., 2014, Li et al., 2014, Yan et al., 2013). They are not able to detect individual crops and crop sequences without expert knowledge of the local to regional agricultural practices or ground-truth data (Bégué et al., 2018). On the individual crop level, only paddy rice agriculture can be identified from short-wave infrared satellite images as it resembles a distinct temporal pattern of flooding / rice transplanting period, growing period and fallow period after harvest (Xiao et al., 2005, Xiao et al., 2006). Also radar imaging systems have proved to be well suited for mapping rice areas with the advantage of not being limited by cloud coverage in rice-growing areas (Bouvet et al., 2009). Other annual crops or cropping systems can often only be identified with additional information, such as from crop calendars, high-resolution imagery, field data, expert knowledge or other ground truth data. Crop identification from satellite images is challenging due to trade-offs between spatial detail and area coverage. Also small field sizes require medium to high-resolution images but these often lack temporal detail (Ozdogan, 2010). As a consequence, signals derived from remote sensing data often represent a mixture of crop types that require temporal ‘un-mixing’ or multi-temporal approaches for mapping multiple crops at the same time (Serra and Pons, 2008, Vyas et al., 2005, Zhong et al., 2015) but these approaches have not been applied on larger spatial scales yet. On the global scale, cropping or harvest intensity, the ratio of area harvested and physical area, has been mapped using national cropland data from the FAO (Ray and Foley, 2013) and gridded cropland data (Siebert et al., 2010). Other approaches for regional or crop-specific data sets include mixing remote sensing and agricultural census data for India and China (Frolking et al., 2006, 2002), using farmer-reported planting and harvesting dates from household surveys (Waha et al., 2013) and national crop calendars (Laborte et al., 2017).

This research has advanced our understanding of the spatial pattern of cropping intensity either for a specific cropping system, e.g. a rice-based system, or for overall cropping intensity or agricultural growing seasons in an individual world region or country but they either lack detail on the crop type and cropping systems or applicability on the global scale.

## 2 Material and methods

**Fig. 1:**
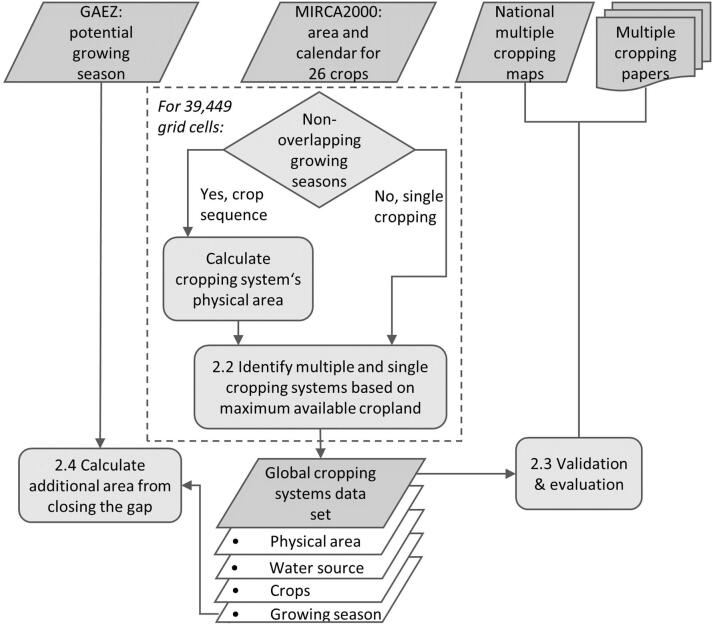
Workflow with inputs, processes and outputs for developing and validating the global gridded cropping systems data set and its application for estimating the global area available for increasing the cropping intensity. Numbers in boxes refer to heading numbers in the materials & methods section of the manuscript.

We develop a method for mapping multiple cropping systems globally (Fig. 1) and present the first global gridded data set of physical area and growing seasons of more than 200 different double and triple cropping systems. Our approach focuses on sequential cropping where two or more crops are grown in a sequence on the same field and one cycle takes 12 months or less to complete. The succeeding crop is planted or transplanted after the preceding crop has been harvested, thus, crop intensification is in the temporal dimension only. We exclude intercropping from the analysis, where crops are grown simultaneously at the same piece of land as well as crop rotation where one cycle takes several years to complete.

The distinction between physical area (sometimes referred to as ‘area sown’, ‘net sown area’ or ‘cropland extent’) and harvested area (sometimes referred to as ‘gross cropped area’ and ‘area under cultivation’) is crucial in our study and not always made clear in the literature and agricultural statistics. Harvested areas can be smaller than physical areas when parts of the crop fields were not harvested, destroyed or left fallow during the growing season or larger when multiple crops are sown in sequence during one growing season. We exclude fallow land from our analysis, i.e. if a field is sown with rice and then left fallow after the harvest, the harvested area is equal to the physical area in our study in the absence of other factors reducing the harvested area.

The ratio between harvested area and physical area is cropping intensity, sometimes referred to as harvest frequency (e.g. in Ray and Foley, 2013). Cropping intensity can also be measured as the number of harvests and referred to as the multiple cropping index (e.g. in Zuo et al., 2014) or cropping frequency (e.g. in Estel et al., 2016), for example as identified from time series of vegetation indices in remote sensing studies. In global studies these measures are aggregated to an average per grid cell of a certain spatial resolution. It is currently unknown which combination of crops and cropping systems exist in a grid cell to lead to a specific cropping intensity. Multiple cropping is characterized by a cropping intensity higher than one and several crops that are grown together, either mixed in space (intercropping) or time (sequential cropping, crop rotation). A multiple cropping system needs to be described by the number of crops, crop types and their growing period as naming only the crops does not allow to distinguish between sequential cropping and intercropping.

### 2.1 Global crop calendar and crop areas

We use data on monthly crop-specific growing areas around the year 2000 (1998–2002) for 26 crop groups as reported by MIRCA2000 (Portmann et al., 2010). MIRCA2000 uses global cropland extent and crop harvested areas (Monfreda et al., 2008, Ramankutty et al., 2008) and also takes (sub-) national agricultural statistics from 402 administrative units into account. The data set provides crop-specific planting and harvest dates using crop calendars from the FAO Global Information and Early Warning System, United States Department of Agriculture and the International Rice Research Institute. MIRCA2000 combines information on crop harvested areas, irrigation, cropping intensities and growing seasons from satellite images and agricultural censuses aiming to minimize errors from inconsistencies between input data and using very little spatial modelling to distribute crop areas across global cropland. MIRCA2000 is one of the only two global gridded cropland data sets that report monthly growing areas and the most recent data set. The alternative global crop calendar data set (Sacks et al., 2010) does not distinguish between rainfed and irrigated crops and is therefore less suited for our analysis here.

The total physical and harvested areas in MIRCA2000 are 1.60 billion hectares and 1.31 billion hectares, respectively with 24% of the total harvested area in irrigated agriculture. Compared to other estimates of global cropland total physical land in MIRCA2000 is<5% below the 1.66 billion hectares reported in (Fritz et al., 2015) and 4% and 6% above the 1.53 billion hectares reported as ‘Arable land and permanent crops’ in FAOStat. An average 0.44 billion hectares are estimated to be left fallow every year (Siebert et al., 2010) and the global average cropping intensity is 0.82 including fallow land (1.31 billion ha / 1.60 billion ha) and 1.13 excluding fallow land (1.31 billion ha / 1.16 billion ha).

MIRCA2000 distinguishes between 26 crop groups and up to five different ‘sub-crops’ for some crops that reflect different crop types, varieties and multiple cropping systems: wheat, barley, rye, maize, cassava, rice and the group of other annual crops. For wheat, rye, barley and maize, spring and winter varieties are distinguished. For rice three varieties are distinguished; upland rice growing 7–8 months, deep water rice growing 7 months and paddy rice, with up to three growing periods typically 4 months long each (Portmann et al., 2010). The other crops or crop groups are millet, sorghum, soybean, sunflower, potato, sugar cane, sugar beet, oil palm, rapeseed, groundnut, legumes and pulses (pulses), citrus, date palm, grapes, cotton, cocoa, coffee, and fodder grasses.

To simplify the presentation of the results, we sometimes aggregate individual crops to crop groups. Millet, sorghum and maize are C4 cereals. Barley, rye and wheat are C3 cereals and all other crops except for rice are grouped together as ‘Others’.

### 2.2 Method for identifying multiple cropping systems and calculating physical cropping system area

The general approach for identifying sequential cropping systems and their respective area share includes the following steps:

1. *Identify possible systems*. Crops with non-overlapping growing seasons can be combined into double and triple cropping systems. Calculate physical cropland for possible crop combinations as the minimum area of all crops in a system. We exclude crop combinations that are deemed technically infeasible or unreasonable, such as paddy rice with upland rice or double cropping with sub-crops that represent different crop varieties not grown consecutively on the same field. We permit only the crop combinations listed in Appendix Table A1.
2. *Ranking and selection*. If in a grid cell there are several crop combinations possible, we rank the systems by cropping frequency (from triple to single cropping) and then by physical area of the cropping system.
3. *Disaggregate total crop area into systems area*. Starting with the first system from list in step 2, we subtract the system’s area from the total available physical crop area in the grid cell and if there is still physical crop area unallocated, we continue with the next system.

**Fig. 2:**
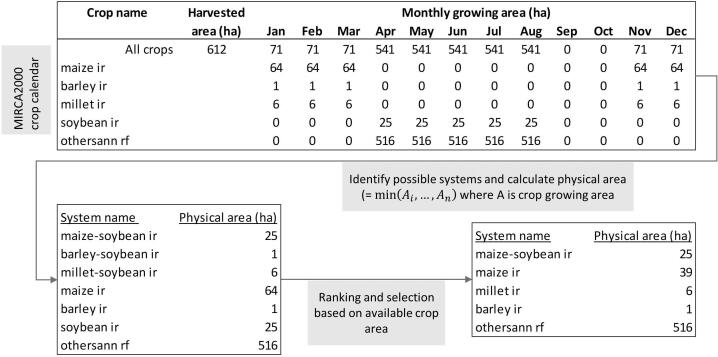
Illustration of the method for identifying cropping systems in a grid cell with multiple non-overlapping growing periods and a cropping intensity greater than 1. In this example the total harvested area is 612 ha, the total physical area is 541 ha and the cropping intensity is 1.13. ir is irrigated, rf is rainfed, othersann is short for others annual, a group of crops as defined in MIRCA2000.

This is done for each of the 39,449 30 arc min land grid cells separately and for rainfed and irrigated systems separately because cropping calendars are based on different data sources and can therefore not be mixed (Portmann et al., 2010). Fig. 2 illustrates the method for an example grid cell. In this example there are three possible double cropping systems with soybean, but maize-soybean is selected as the only double cropping system because it has the largest physical area as a system. As the maize area exceeds the soybean area, the remaining maize area is assumed to be the physical area of a separate field with maize as a sole crop. All remaining crops are grown as sole crops and the sum of the individual system’s area matches the total available physical cropland in the grid cell.

This means we disaggregate the total available physical cropland in each grid cell into areas of single cropping, double cropping and triple cropping systems. If the sum over all available systems’ physical area exceeds the total available cropland in a grid cell, some cropping systems are excluded, and we therefore underreport the total physical cropland without fallow land slightly by 0.3% globally, 1,154 million hectares (Mha) instead of 1,158 Mha. Where several crop combinations are possible, we choose the one with the largest physical system area and exclude the others. This mostly happens in areas with high cropping intensities and where a large variety of crops is grown such as in the Andean countries, East and Southeast Asia and Central and Eastern Africa. In 43% of all grid cells all possible cropping systems were allocated to total cropland in the grid cell and in another 23% of all grid cells one to ten alternative cropping systems were not included. In the example above millet-soybean and barley-soybean are not included as potential double cropping systems as soybean is assumed to be grown together with maize.

Cropping systems with different water sources per crop or season are not represented in the final data set and each cropping system is assumed to be either irrigated or rainfed. Rainfed cropping systems with a fully or partially irrigated second crop, as e.g. in some rice–wheat systems in the eastern part of the Indo-Gangetic belt, in Bangladesh, and parts of the Indian states of Bihar and West Bengal are represented as either rainfed or irrigated.

It is important to note that with the method outlined above we are able to identify multiple cropping systems with two or three crops grown in a sequence during a 12-month period, but not crop rotations that occur over several years or inter-cropping systems in which more than two crops are grown simultaneously or with overlapping growing periods in the same field. We however capture sequential cropping systems with two or more crops grown in the same as well as in two distinct rainy seasons (in areas with a bimodal rainfall pattern). We aim at representing such multiple cropping systems at a 30-arc min resolution that are realistic considering climate, crops and crop varieties currently cultivated and management currently practiced but not necessarily the most important cropping systems in economic terms. Other factors such as farmer’s attitude towards risk, access to fertilizer and seeds, soil quality, the long-term sustainability of a cropping system, availability of labour, market prizes and periodical large-scale climate pattern such as the El Nino Southern Oscillation will affect farmer’s choices to cultivate a second or third crop. Also the analysis is limited to identifying the area share of a particular cropping system in 30 arc minute grid cells, not crops or cropping systems of different plots of land or farmer’s fields. Only where field sizes are large and the diversity of crops and systems is low this distinction might be irrelevant.

### 2.3 Validation and evaluation

A formal validation of our global multiple cropping data set is not possible due to the lack of global data to compare to but we take the following steps to evaluate accuracy for selected countries, and describe potential uncertainties and limitations in the data set: (i) review of the literature describing specific cropping systems in different world regions (N = 33, Appendix Table A2) and comparison to systems found in our data set, (ii) listing known issues related to the input data and method used, (iii) grid cell-to-grid cell comparison with national gridded data sets available for China and India, (iv) comparison of total multiple cropping area extent reported in literature and agricultural census statistics and (v) assess the number of alternative cropping systems to the ones identified in the final data set.

For China and India we compare our global multiple cropping maps with two independent maps of multiple cropping systems (Frolking et al., 2006, 2002) and discuss differences and similarities in the spatial patterns. For China, Frolking et al. (2002) distinguish between 47 rainfed and irrigated rice and non-rice cropping systems with 18 different crops - alfalfa, beet, maize, rapeseed, soybean, vegetable, cotton, potato, rice, fallow, millet, non-legume hay (non-legume crop for hay), oat, wheat, sorghum, sugar cane, sunflower and tobacco. For India, Frolking et al. (2006) distinguish between 54 rainfed and irrigated rice cropping systems with 15 different crops - fallow, pulse, oilseed, groundnut, soybean, potato, vegetable, wheat, fiber, pulse, barley, maize, sorghum and sugar cane. For a comparison between these two national maps and our study, we use the 32 crops common in all three data sets and group 24 of them into 5 crop groups:

– Cereals: millet, sorghum, maize, barley, rye, wheat, oat
– Oil crops: oil palm, soybean, sunflower, groundnut, rapeseed, oilseed
– Root crops: cassava, potato, sugar beet, beet
– Others perennial crops: citrus, date palm, grapes, sugar cane, other perennial crops
– Fodder grasses: alfalfa, fodder grasses

The following groups and individual crops were not grouped: pulse, rice, cocoa, coffee, cotton, tobacco, others annual and vegetables. Fallow and non-legume hay are not considered as crops in MIRCA2000 and are therefore excluded from the comparison.

A binary classification test between our dataset and the national data set is difficult as they were developed at a different spatial scale and for different, but close baseline years and in both, the national and the global data sets several cropping systems exist in a grid cell. We check whether the system with the largest area share in the national data set was identified as a potential cropping system in the global data set, evaluate the spatial pattern of single, double and triple cropping in China and India and discuss agreement and disagreement to our global data set nonetheless as it can be an indication of the appropriateness of the developed method and data set presented here.

### 2.4 Cropping intensification potential

We calculate the potential for increasing cropping intensity on current rainfed and irrigated cropland from the area and growing season of actual single cropping systems and the potential growing season for rainfed farming. The potential growing season is the number of consecutive months with favourable temperature and soil moisture conditions as defined in the FAO-AEZ framework (van Velthuizen et al., 2007). The original data available at the FAO’s geospatial data server GeoNetwork (FAO, 2007) is based on 16 classes each class 30 days wide, except the first and the last three classes and we converted them into 12 classes representing the length of the potential growing season in months (Figure S1). We first calculate the difference between the potential and actual growing season and then sum over all suitable single crop areas with a minimum difference according to assumptions about the minimum length of a second crop cycle (Madamba et al., 2006) and the number of months required for a short fallow period and time to prepare the land before the next year. The scenarios used are:

- The difference between potential and actual growing season is at least two months for growing a second crop (referred to as A2 scenario).
- The difference between potential and actual growing season is at least four months for growing a second crop with a minimum growing season of two months and leaving the land fallow for at least two months while it is prepared for the next cycle (referred to as A4 scenario).

In two additional scenarios we restrict the calculation of potential crop areas to regions with low risk of crop failure due to frost or drought as farmers might tend to avoid the risk of losing the second harvest. Low frost risk is defined as minimum mean monthly temperature being higher than or equal to 10 °C. Low drought risk is defined as the coefficient of variation of annual rainfall being less than the global average of 19% which indicates that the mean annual rainfall is fivefold the standard deviation.

- As first scenario above but with reduced frost and drought risk as specified (referred to as A2- scenario).
- As second scenario above but with reduced frost and drought risk as specified (referred to as A4-scenario).

Mean monthly temperatures over the time period 1970 to 2000 are taken from WorldClim version 2 (Fick and Hijmans, 2017) and the coefficient of variation represents the year-to-year variability in 1980 to 2000 as reported in the AgMERRA climate forcing data set for agricultural modelling (Ruane et al., 2015). The potential for cropping intensification is then expressed as potential crop area increase on current cropland in hectares and percentages of current single cropping area and current harvested area.

## 3 Results

### 3.1 Global multiple cropping area and systems

**Fig. 3:**
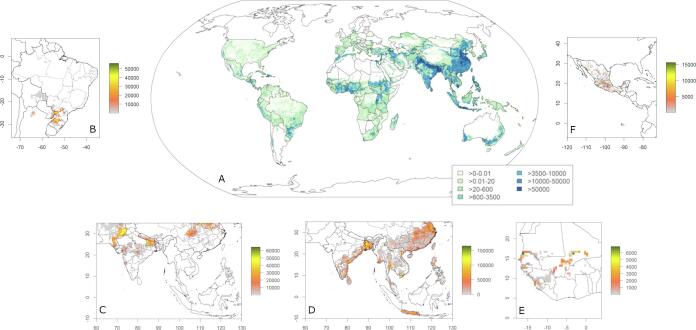
**Physical area (hectare) of multiple cropping systems per 30 arc-min grid cell, 1998**–**2002.** A Global multiple cropping area. B Rainfed soybean-wheat double cropping system in South America. C Irrigated wheat-rice and rice-rice (D) double cropping system in South, East and Southeast Asia. E Irrigated rice-rice double cropping system in West Africa. f Irrigated maize-wheat double cropping system in Central America. White areas indicate locations with total crop area less than or equal to 1% of the grid cell area.

We estimate that 134.4 Mha land is under multiple cropping, 12% of global cropland (Fig. 3). 5% of global rainfed cropland and 40% of global irrigated cropland is under multiple cropping (Table 1). Our results suggest that double cropping extents to 130.4 Mha (11.3%) with 81.5 Mha irrigated and triple cropping extents to 4.1 Mha globally (0.35%) with 2.9 Mha irrigated. Single cropping extents to 1020.6 Mha land (88.4%) with 132.1 Mha irrigated cropland. This amounts to 1.16 billion hectares to represent the global physical cropland extent without fallow land. Together with global fallow land of 0.44 billion hectares (Siebert et al., 2010) this amounts to global physical cropland of 1.60 billion hectares which equals the global cropland extent presented in Portmann et al. (2010) which was used as input data here and is well within the range of other estimates of global cropland extent of 1.22 to 1.71 billion hectares (Ramankutty et al., 2008). Total harvested area is 1.29 billion hectares and global average cropping intensity is 0.81 including fallow land and 1.1 excluding fallow land. The average cropping intensity in a grid cell as estimated in MIRCA2000 was already presented in earlier studies (Portmann et al., 2010, Siebert et al., 2010) so we do not show it again here.

**Table 1:** Global physical cropland of major cropping systems (in million hectares).

Crop / Crop group	Rainfed Area	Irrigated Area	All cropland
Single cropping			
*With…*			
Rice	49.40	13.19	62.59
C3 cereal	194.37	27.68	222.05
C4 cereal	172.61	16.62	189.23
Other	472.10	74.58	546.68
*Sub-Total*	*888.48*	*132.07*	*1020.55*
Double cropping			
*With…*			
Rice	8.68	34.05	42.73
C3 cereal	9.94	12.02	21.96
C4 cereal	11.21	3.05	14.26
C3 / C4 cereals	2.63	13.94	16.57
Rice / C3 cereal	0.57	14.59	15.16
Rice / C4 cereal	2.26	0.80	3.06
Other	13.51	3.10	16.61
*Sub-Total*	*48.80*	*81.55*	*130.35*
Triple cropping			
*With…*			
Rice	0.41	2.51	2.92
C3 cereal	< 0.01	< 0.01	< 0.02
C4 cereal	0.19	0.26	0.45
Rice / C3 cereal	< 0.01	< 0.01	< 0.02
Rice / C4 cereal	0.25	< 0.01	0.26
Other	0.26	0.13	0.39
*Sub-Total*	*1.15*	*2.91*	*4.06*
Total	**938.43**	**216.53**	**1154.96**

Globally, we find more than 200 different multiple cropping systems as a combination of 25 annual crops. The 29 multiple cropping systems with the largest crop area globally account for 80% of the global multiple cropping area. Cereals harvested only once a year in temperate areas with or without irrigation are the most important cropping system with respect to area coverage. Double cropping is important in tropical and subtropical agriculture with double cropping of cereals and a combination of cereals and pulses and cereals and oil-crops being the most important. 44% (49.63 Mha), 13% (24.12 Mha) and 10% (13.49 Mha) of the rice, wheat and maize area, respectively are under multiple cropping, making it an important cropping system for cultivating these crops (Appendix Table A3). The top five multiple cropping systems with largest physical area globally are: irrigated rice-rice (32.66 Mha), irrigated double cropping with wheat and rice (14.38 Mha) irrigated double cropping with wheat and maize (12.16 Mha), irrigated double cropping with wheat and cotton (4.3 Mha) and rainfed double cropping with rapeseed and another annual crop (3.56 Mha). Together with monocultures of wheat, maize, rice, soybean and pulses (468.8 Mha) these systems account for more than 50% of the global cropland extent. On 70% of the area under multiple cropping the system consists of different crops, 30% are monocultures with the same crop harvested more than once.

### 3.2 Regional importance of multiple cropping and regional validation

**Fig. 4:**
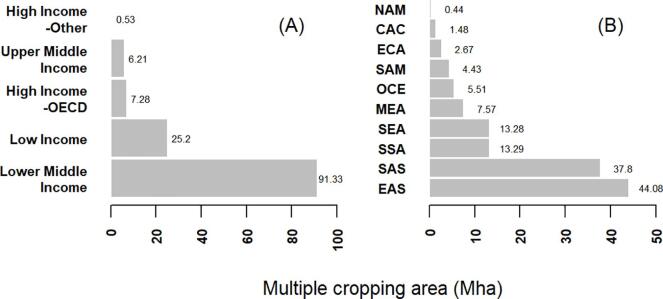
**Multiple cropping area by income group (A) and geographical region (B).** The codes for the geographical regions (B) are based on World Bank country groups: NAM North America, CAC Central America and Caribbean, ECA Europe and Central Asia, SAM South America, OCE Oceania, MEA Middle East and North Africa, SEA Southeast Asia, SSA Sub-Saharan Africa, SAS South Asia, EAS East Asia. The grouping by income (A) is based on the gross national income (GNI) per capita as defined by the World Bank.

Multiple cropping mostly occurs in tropical and subtropical climates with a rainy season long enough or adequate irrigation to grow two or three crops in a sequence during one agricultural year. The majority of multiple cropping areas are located in East Asia (44.1 Mha, 34%) and South Asia (37.8 Mha, 29%), and in the low to lower middle income countries (116.5 Mha, 86%) (Fig. 4).

For the two world regions with largest multiple cropping area share, East Asia and South Asia, we compare total multiple cropping area and spatial pattern of cropping systems in our global data set to two national data sets for China and India. For other world regions we evaluate the accuracy of the global data set by comparing to multiple cropping area estimates and multiple cropping system descriptions in the literature.

Multiple cropping systems in China and South Asia

The grid cell-by-grid cell comparison indicates that in 70–75% of all 30 arc min grid cells the reported cropping system with the largest physical area in the national data set was identified in our global data set as well (Fig. S2-3). In the remaining grid cells, the system identified can be very similar to the observed, e.g. for the province of Jiangsu in China, the national data set reports irrigated rice-cereals as the dominant system whereas we found irrigated rice-rice to be dominant. The spatial pattern of single and double cropping in *China* as well as total cropland is reproduced very well, except for double cropping in the Northeast (provinces Liaoning, Jilin, Heilongjiang) (Fig. S4-5). In *India*, the spatial pattern of the systems with largest crop area in the national data set is reproduced very well, except for parts of the states of Rajasthan and Gujarat in western India and the state of Uttar Pradesh in northern India. The rice-cereals irrigated cropping system known to be important in North India is not represented very well (Figure S7) because an additional cropping period for rice (in winter season) and wheat (in summer season) was introduced in MIRCA2000 that deviated from the FAO crop calendar for these states in order to accommodate the irrigated harvested area exceeding the available area equipped for irrigation (Portmann et al., 2010). Therefore, our data set reports irrigated double cropping with cereals, pulses, oil crops or irrigated triple cropping with rice as prevalent in parts of Uttar Pradesh, Punjab and Haryana.

**Fig. 5:**
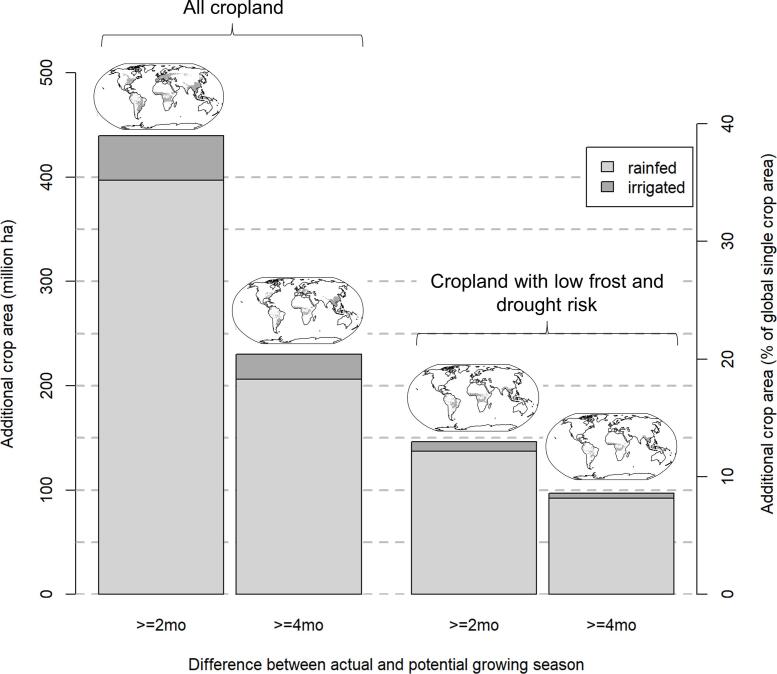
**Potential for increasing cropping intensity on current global croplands**. Each bar shows additional crop area measured as total hectares and percentage of global single cropping area (=1.02 billion hectares) in a different scenario: with two- or four-months difference between potential and actual growing season, for all cropland and for cropland with low frost and drought risk. Maps show the respective geographic areas with more than 1000 ha additional cropland available. See Figure S8 for larger maps of the four inset maps and Appendix Table A9-10 for area statistics by shown here and by World Bank country group.

Cropping intensity increases from the north-west to the south-east in *China* with single cropping being prevalent in Northwest China and Northern China and multiple cropping occurring in the Huanghe-Huaihe-Haihe plain and in the middle and lower areas of the Changjiang (Yangtze) river (Yan et al., 2013). We estimate the total multiple cropping area and area share in China as 43 Mha (36%). This is close to the 50 Mha (38%) reported previously (Frolking et al., 2002) (Appendix Table A4). There is uncertainty in the proportion of cropland cultivated with triple cropping in China; the estimates range from 1.8% (Yan et al., 2013) to 10.9% (Frolking et al., 2002, Qiu et al., 2003), depending on the data source and base year and is 0.3% in this study. Also in other maps of Chinese cropping systems for 2006/2007 (Li et al., 2014), triple cropping is not as prevalent in South China as shown in Frolking et al. (2002) (Frolking et al., 2002). Multiple cropping is generally less important in Northern China but exists in Northeast China and the North China Plain (Qiu et al., 2017, Zuo et al., 2013).

Multiple cropping systems in *South Asia* are mostly based on rice. Rice and other crops can be grown in up to three seasons, known as *kharif* (June-October), *rabi* (November-February) and summer (March-May) seasons. The *kharif* season is the primary season and relates to the north-west monsoon season, and the *rabi* season relates to the south-east monsoon season (Frolking et al., 2006, Gumma et al., 2016). Triple cropping with crops grown in all three seasons only occurs on 3% of the cropland in *India*, mostly in the eastern and southern states of Kerala, West Bengal and Tamil Nadu, Assam and Bihar (Frolking et al., 2006). We estimate the total multiple cropping area and area share in India and South Asia as 24 Mha (16%) and 37 Mha (21%), respectively. This is lower than the 44–58 Mha (35–41%) and 103 Mha (49%) reported in literature and official censuses (Appendix Table A4, Appendix Table A5). The total multiple cropping area of rice-only systems and area share in India and South Asia is 14 Mha (44%) and 23 Mha (50%) which agrees well with the 17 Mha (42%) and 25 Mha (48%), respectively reported elsewhere (Appendix Table A6, Appendix Table A7). While the total rice area harvested in India and South Asia is estimated correctly, the double cropping areas with rice and other crops are underestimated and the single cropping area extent overestimated, very likely because of the misrepresentation of the growing season of crops other than rice in the national crop calendars used here. Further, there are five to ten years between the two estimates which could explain the disagreement as well. The multiple cropping area in India has increased from 44.0 Mha in 2000–01 to 51.6 Mha in 2005–06 (+17%) and 56.0 Mha in 2010–11 (+27%) (Government of India, 2017) which explains part of the underestimation in non-rice double cropping areas in the global data set. In the Punjab region, at the border between India and Pakistan, the area and growing season of the wheat-cotton cropping system is represented well in our data set. Cotton is picked at the latest from the beginning of December to the first week in January and followed by a wheat crop grown in the *rabi* season (Sheikh et al., 2003). The total rice–wheat area in India which elsewhere is reported to be between 8.8 Mha and 9.2 Mha (Frolking et al., 2006, Yadvinder-Singh et al., 2014), is underestimated in our data set (2.4 Mha) with larger areas of rice in other systems. In the western Indo-Gangetic plains, states Punjab and Haryana, the multiple cropping area is too low and the single cropping area too high (Appendix Table A8). The rice–wheat areas in *Pakistan, Bangladesh* and *Nepal* agree well with other estimates: 2.8 Mha, 0.38 Mha and 0.37 Mha compared to 2.2 Mha, 0.40 Mha and 0.57 Mha, respectively (Yadvinder-Singh et al., 2014).

## 4 Multiple cropping systems in Latin America

Cropping systems with soybean as the main crop and a so-called *safrinha* crop cultivated as a minor crop in the same year is common in *Brazil* (Anderson et al., 2017) where the rainy season is long enough. Commonly this is a double cropping system with maize, cotton or millet as *safrinha* crops e.g. in the state of Mato Grosso. Soybeans planted in September and harvested in February (early harvest) or at the latest in early April (late harvest) are followed by maize (planted in February), millet (planted in March-April) or cotton (planted in January) (Arvor et al., 2011). Cropping intensity has not always been that high. During just 14 years (2001–2014), soy production shifted from predominantly single-crop systems to majority double-crop systems (Kastens et al., 2017). In Mato Grosso double cropping areas with soybean increased from 500,000 ha (15% of cropland) to 3 million ha (50% of cropland), between 2001 and 2011 (Spera et al., 2014). The net cropped area cultivated with double cropping systems including two commercial crops increased from 6% to 30% between 2000 and 01 and 2006–07 (Arvor et al., 2012). In 2006–07 double cropping accounted for 30% of the total planted area of 5.6 Mha in Mato Grosso (Arvor et al., 2011). Most of the double cropping area is found in South and Central Brazil, where e.g. in the state Rio Grande do Sul at the border to Uruguay, soybeans are planted after the wheat harvest in December. Other double cropping systems in South Brazil are dry and wet season peanuts (e.g. in São Paulo and Paraná), dry and wet season beans or two harvests of potatoes (Dalrymple, 1971).

Double cropping is also common in *Mexico*, especially in the Pacific North region (states of Sonora and Sinaloa) and in the Central region (Guanajuato) (Dalrymple, 1971). Wheat is grown as a winter crop followed by maize in summer (Limon-Ortega et al., 2006). In *Argentina*, ~30% of the soybean was sown as double crop after harvest of winter wheat” (Monzon, 2009) which presumably reflects the situation in or before 2008/2009 but the share might have been even smaller in 2000. In the Buenos Aires province wheat-soybean double cropping has been practices since the late 80 s (Andrade and Satorre, 2015), however the actual area share of wheat-soybean systems is unknown.

We find many of these systems in the global data set as well, except the soybean-maize and soybean-cotton system in Mato Grosso, probably because the data set refers to the year 2000 and there have been large increases in cropping intensity since then. Double cropping in Mato Grosso in the data set is represented only by small areas of soybean-pulses and soybean-wheat systems. The wheat-soybean system can also be found in southern Brazil with soybean planted in November and wheat harvested in October. Other systems prevalent in South and Central Brazil in the global data set are soybean-pulse in Goiás and maize-pulses in Minas Gerais with the first crop planted in November and the second crop harvested in October. The wheat-maize system prevalent in Mexico seems to be represented well in the global data (Fig. 3), it’s the second largest cropping system in Mexico with wheat planted in November. We estimate the multiple cropping area in Brazil, Mexico and Argentina as 2.8 Mha, 0.7 Mha and 75,000 ha.

## 5 Multiple cropping systems in Africa

Multiple cropping systems in Africa are based on maize, wheat, millet or sorghum, e.g. maize-legumes in *Ethiopia* (Teklewold et al., 2013), wheat-maize and groundnut-maize in *Southern Africa* (Waha et al., 2013), and wheat grown during winter and maize during summer under irrigation in *Egypt* (Ouda et al., 2015). Irrigated rice farming with two rice harvest is common in inland river valley of the West African Sahel and *Madagascar* (Laborte et al., 2017). In Central and Northern Ethiopia maize, wheat and sorghum are grown during the main rainy season, *kiremt* from June to September which can be followed by a second crop, maize, teff or sorghum in the short rainy season *belg* from March to May if there is enough rainfall (Kassie et al., 2013). In *Tanzania*, the main growing season *masika* from March to May is followed by a short growing season *vuli* from October to January, in particular in North Tanzania. Tobacco, beans, cowpeas and wheat are mostly grown in the short season, sometimes under irrigation. Maize, millet, sorghum and paddy rice are grown mostly in the long rainy season (Tanzania Ministry of Agriculture, 2012). This bimodal rainfall pattern permitting double cropping is prevalent in other parts of East Africa, with one season in March-May and a second season in September/October to December (Mutai and Ward, 2000). These systems are represented in the global data set as well with similar planting and harvesting months for the irrigated wheat-maize systems in *Egypt* and the irrigated double cropping rice system in *Madagascar* (first planting in November/December). The first planting of rice in West African river valleys is two months earlier (November instead of January) than reported in a recently published rice atlas (Laborte et al., 2017). For the remaining multiple cropping areas of Africa, we can assess the plausibility of double cropping based on the length of growing period and the occurrence of a bimodal rainfall pattern. Two distinct rainy seasons can be found in *Somalia*, southern *Ethiopia*, northern *Kenya*, the Niger delta and the Guinea coast and the growing period is longer than five months in southeast *South Africa* (Lesotho highlands, Kwazulu-Natal, Eastern Midlands), and West and Central Africa south of 12°N (Liebmann et al., 2012, Vrieling et al., 2013). For the areas with bimodal rainfall pattern in Africa, it’s important to consider that there is a considerable variability in the occurrence and onset of the rainy seasons (Dezfuli and Nicholson, 2013, Nicholson and Dezfuli, 2013, Segele and Lamb, 2005), so even though we identify a multiple cropping system it might not be feasible every year. We estimate the multiple cropping area in Egypt, Ethiopia, South Africa, Tanzania, Madagascar and Kenya as 2.1 Mha, 1.9 Mha, 0.69 Mha, 0.56 Mha, 0.48 Mha, and 0.24 Mha, respectively. The total multiple cropping area in Africa in our study is 16.1 Mha, which is 11% of the total physical crop area of 147 Mha. The area share agrees well with a recent estimate for 2014 where double cropping area is 12% of the total physical crop area (Xiong et al., 2017), however, both the double cropping and total crop area are larger in 2014. While we identify several cropping systems and their physical area in each 30 arc minute grid cells, it is likely that there is a higher diversity of cropping systems on the farm scale, in particular in smallholder agriculture and where plot sizes are small.

The agreement between our global dataset and the national datasets depends largely on identifying irrigated areas and irrigated cropping sequences correctly as most of them would be multiple cropping systems. In China and India 70–75% of the multiple cropping area is irrigated. Other reasons for disagreement are the difference in baseline years, misrepresentation of the growing season of crops other than rice in the national crop calendars for Asia used here, uncertainty about the area definition in agricultural statistics (area harvested or physical area). Other known issues of the input data used were described before (Monfreda et al., 2008, Portmann et al., 2010, Siebert et al., 2010) and are relevant for the representation of multiple cropping systems in our study. These include the underrepresentation of minor crops, crop varieties and cropping systems that can be important locally, limited spatial resolution of crop calendars, simplification of local and regional agricultural practices in macro-scale data sets, spatial disaggregation from national agricultural inventory data to grid cells considering satellite-derived land cover maps, merging of a remote-sensing based and a ground based observation systems and different definitions of cropland in different data sets.

### 5.1 Potential for increasing global crop area

As increasing cropping intensity has been an important strategy in the past for increasing harvest area and crop production without expanding physical cropland, we further calculate the potential for increasing cropping intensity on cropland with currently just a single harvest. This intensification potential only refers to the temporal, not the spatial dimension of mixing crops in cropping systems. Based on our crop-specific multiple cropping dataset and different intensification scenarios that reflect the opportunities for growing a second crop and leaving the land fallow for a short period of time before the next agricultural season, we find that a maximum of 395 Mha of global crop area in 2000 is available for this type of intensification (39% of global single cropping area). This is only possible in a scenario with a second crop growing for two months such as pulses or legumes but without any short fallow period before the next year (Fig. 5). This is 41–46% less than previous estimates that suggest an increase of global harvested area by a maximum of 666 Mha to 736 Mha by increasing cropping intensity (Ray and Foley, 2013, Wu et al., 2018). 207 Mha (20%) are available to grow a second crop if the land is left fallow for two months and the second crop’s growth cycle is just two months. Both these estimates include cropland in areas where frost-occurrence and high rainfall seasonality increase the risk of crop failure. Excluding these high risk areas limits the available land further to 131 Mha (13%) and 87 Mha (9%), in a scenario with second growing seasons of two and four months, respectively (Fig. 5). The potential for increasing harvested area on currently irrigated land is low globally, because growing seasons of irrigated cropping systems are already very close to the potential growing period.

The additional total crop area for intensification is largest in South America (46–57 Mha) and lowest in Oceania (0.8–1.5 Mha) when not considering frost and drought risk (Appendix Table A10). The maximum intensification potential in Europe and Central Asia (76 Mha) and North America (63 Mha) exceeds that of South America but only in the most optimistic scenario of requiring only two months for a second crop cycle. The potential area for intensification declines to less than half and less than one fourth of the maximum for Europe and Central Asia and North America, respectively in the scenario with four months difference between current and potential growing season. In the scenarios that additionally consider that farmers might avoid risking a crop failure in an additional crop cycle where the frost or drought risk is high the additional crop area for intensification is largest in Sub-Saharan Africa (22–40 Mha) which accounts for about one quarter of the global intensification potential in these scenarios and lowest in Oceania (38,700–41,500 ha).

## 6 Discussion and conclusion

This is the first time that multiple cropping systems have been mapped on a global scale, with the crop-specific and area representation that is needed for global assessments of land use and food production, and largely consistent with independent national data sets. This study goes beyond previous work in that we describe multiple cropping systems in terms of the cropping sequence, growing season and physical area of each systems and not just the average cropping intensity. We discuss some uncertainties in the data set itself for specific world regions above. There are also a number of limitations with regards to our estimates of the potential for increasing cropping intensity.

Our estimates of potential harvested area increases are rather optimistic in that cropping intensity in some parts of the World is probably already higher than the global crop calendar indicates due to a lack of sub-national differences in production zones for most countries, except for, Indonesia, Brazil, India, China, Argentina, Australia and the United States of America. Also, a short-duration crop such as a legume or pulse in a cropping system might only be grown as a cover crop or forage crop and that is another source of potential overestimation of the intensification potential. The potential to increasing cropping intensity might further be limited by soil degradation (Gibbs and Salmon, 2015), biotic stresses (Beddow et al., 2015), and the lack of seeds, fertilizer, infrastructure and market incentives (Lambin et al., 2001, VanWey et al., 2013), processing and storage infrastructure for some farmers, technologies and rainfall variability and climate change (Arvor et al., 2014, Cohn et al., 2016, Liu et al., 2013, Yang et al., 2015) just to name a few.

The data we use refers to the time period 1998–2002, missing almost two decades of agricultural intensification in which, potentially, gaps between actual and potential cropping intensity were already reduced. A prominent example is the Brazilian state of Mato Grosso where soybean production has shifted from mostly single-cropped to double cropping in just 14 years between 2001 and 2014 and the area share of all double cropping has increased from 15% to 50% between 2001 and 2011 (Kastens et al., 2017, Spera et al., 2014). In other countries cropping intensity has been rather stable. The double and triple cropping areas in China for example have changed very little from the 2000 s to 2013, by −1.37% and + 0.48%, respectively (Qiu et al., 2017). Globally, harvested areas have increased by 16% between 2000 and 2016 according to FAO statistics. It is unclear how much of this occurred on current or new cropland, but very likely these changes have affected the spatial distribution of single and multiple cropping systems as well.

An important next step will be to update the crop calendar and crop areas used here to a more recent time period. This is a challenging task as several underlying data sets need to be updated first. Most countries conduct an agricultural census every 10 years or even less often and data for the years in between are estimates based on temporal interpolation or from sample surveys. Although the most recent estimate of global cropland extent of 1.561 billion hectares from the Food and Agriculture Organization of the United Nations (FAO) is for the year 2017 this is an aggregate that includes official, semi-official, estimated and calculated data and the global gridded maps of cropland extent are less often updated. To our knowledge the most recent global gridded cropland maps are the unified cropland layer at 250 m resolution for the year 2014 (Waldner et al., 2016) and the global cropland extent map at 30 m resolution developed by the GFSAD30 project for the years 2013–2015 which is not yet publicly available for download but both do not include any crop-specific harvested areas and their growing periods. Even for a country with lots of cropland under multiple cropping like China, as far as we know, the most recent and published area estimate and multiple cropping map is for the year 2013 and was published in 2017 (Qiu et al., 2017).

Despite these shortcomings our findings are novel and of significant relevance for global food production, food security and sustainability research as discussed in the following two final sections.

### 6.1 Implications for global food production and food security

Increasing cropping intensity in suitable areas could increase global harvested areas of currently 1.29 billion hectares by 7–31%, depending on the scenario. Estimating the additional crop production from this second harvest is very difficult as many factors influence crop growth in different growth stages and final yields. Assuming that the yields of the second crop are at most reaching 80% of that of the first crop, which has been reported as an achievable yield (Yadav et al., 2000), would translate into a similar increase of global crop production, <30%. This is much lower than the previous estimates of a potentially 39–50% increase in global production (Mauser et al., 2015, Ray and Foley, 2013). The only way to increase global harvested areas and thus global production by about 50% without expanding physical cropland is to assume that a second crop can be grown irrespective of any climatic constraints and where the current crop cycle is less than or equal than ten months. The additional area would be 740 Mha, an increase of global harvested areas of 57%.

These findings are important because land use intensification could significantly boost production of food, feed and bioenergy crops with positive economic and food security outcomes. Achieving this by increasing the cropping intensity on current cropland could avoid expanding physical cropland but still provide enough food to fulfil the demand of a growing population. Additional cropland needed has been estimated at 10–25% (Schmitz et al., 2014), however this estimate depends strongly on the crop yields achievable in the future. About 1.2 billion to 1.5 billion people live in areas with potential for increasing cropping intensity as defined here. It is however impossible to draw conclusions on the effect of increasing cropping intensity on national or local food security at this stage for three reasons. Firstly, we only present the area of single and multiple cropping systems. Crop yields and overall production from current and new cropland will depend on, among other things, soil conditions, the length of the growing period and crop management. Secondly, although agricultural productivity can be a good proxy for measuring food and calorie availability, availability does not necessarily assure adequate nutrition (Pinstrup-Andersen, 2009). Many countries’ food insecurity is associated with a food access rather than a food availability problem (Smith et al., 2000). There are many facets and causes of food insecurity on different spatial scales and different types of agricultural households face different challenges with respect to the four dimensions of food security: availability, access, utilization and stability and to non-food factors (Carletto et al., 2013). And finally, we only consider harvested annual crops in our analysis, but do not separate into different uses. It is estimated that about 85% of all maize demand and 30% of all wheat demand is for livestock feed and for other uses than for direct human consumption (Shiferaw et al., 2011, Shiferaw et al., 2013).

### 6.2 Increasing cropping intensity in the context of sustainable agriculture

The environmental consequences of introducing a second crop on currently single cropping land depend on the type of crop, management and other local context. The three main implications for environmental outcomes when cropping intensity increases are that (i) the intensification of current cropland leads to positive outcomes for biodiversity elsewhere, (ii) the intensification increases economic incentives to expand current cropland with negative outcomes for biodiversity and (iii) the intensification increases resource use, potentially beyond sustainable levels and

The arguments for the first trajectory are summarized as the ‘Borlaugh hypothesis’, the ‘subsistence hypothesis’ and the ‘economic development hypothesis’ in Angelsen and Kaimowitz (2001). In brief, the expectation is that either higher yields reduce agricultural land, farmers use the land only to a certain point, or higher yields contribute to economic development, reduced poverty, increasing demand for environmental services and decreasing environmental degradation. The areas with highest potential for intensification through increased cropping intensity are in China, South-East Asia and tropical Central and West Africa and Central America and Brazil. They are located in hotspots of biodiversity or high tree cover, e.g. Brazil’s Cerrado, Madagascar, the West African forests, Indo-Burma and the Malay Peninsula, Borneo, Java and Sumatra (Hansen et al., 2013, Myers et al., 2000) and increasing cropping intensity could avoid the need to expand cropland into natural ecosystems, however this land sparing effect is highly debated and depends on local circumstances and effective environmental governance (Angelsen and Kaimowitz, 2001, Ewers et al., 2009, Morton et al., 2006, Phalan et al., 2016). As we only present the global picture here, a separate analysis for each of these regions needs to be conducted for understanding the potentials and limitations of increasing cropping intensities regionally as has been done e.g. for China (Yu et al., 2017, Zuo et al., 2014).

Growing a second crop might require more resources, either in terms of labour, energy, water, nutrients, agro-chemicals or all the above. Therefore, the possible implications of an intensification scenario are that potentially either the second crop needs to be partly or fully irrigated, there is no fallow period to restore soil fertility, and crops need to grow in a semi-controlled or fully controlled environment such as in a greenhouse which increases energy consumption or a combination of these. Water scarcity is already high in many areas with high population densities, high rates of water extraction or low water availability so very likely water withdrawals cannot be increased as much as needed to support increases in cropping intensity in the future. Some of these consequences can already be observed for the example of rice–wheat cropping systems in South Asia and include declining water tables, ground water pollution, loss of genetic diversity and disease and pest outbreaks (Bhatt et al., 2016). Apart from the direct environmental damage, these unintended consequences of intensification can endanger the long-term productivity and profitability of the cropping system. However, these problems are not specific to sequential cropping systems but to intensively managed systems when incentives to overuse fertilizer, pesticides and water are high. One example of ‘ecological intensification’ that included growing a second crop is the soybean system in Mato Grosso where farmers cultivate a second non-commercial crop to prevent soil erosion, break pest cycles and enable the adoption of no-tillage practices (Arvor et al., 2012). Another implication of introducing a second crop into a system, this diversification effect benefits ecological functioning and biodiversity on the farm but this depends largely on the plant composition and structural organization at the field scale (Gaba et al., 2015, Tscharntke et al., 2012).

## 7 Data and code availability statement

The multiple cropping data set was created as an R *raster* object with multiple layers, and is available as NetCDF files, together 128 MB large and available on CSIRO’s data repository (https://data.csiro.au/, see data citation below). A preview of the data set is available at geo-wiki.org. The data set was designed to be used together with other global gridded data sets or global crop, agricultural and Earth System models for further analysis that could include (i) quantifying the amount of food produced in multiple cropping systems, (ii) studying climate change impacts on crop production in multiple cropping systems globally, and (iii) studying the biogeochemical interactions and water and carbon fluxes between soil, crop, and the atmosphere in multiple cropping systems. The R code for generating the global gridded multiple cropping systems data set is available through an R package *multicrop* deposited at CSIRO’s code share repository (https://bitbucket.csiro.au/projects/MULTICROP) and revisions of the source code are managed there and are available on request.

## 8 Data citation

Waha, Katharina; Dietrich, Jan P.; Portmann, Felix T.; Siebert, Stefan; Thornton, Philip K.; Bondeau, Alberte; Herrero, Mario (2020): Multiple Cropping Systems of the World. v1. CSIRO. Data Collection. https://doi.org/10.25919/5f1f7bb3270bb.

## Author contribution

KW designed the research, wrote the paper and performed the data analysis in close collaboration with JPD, SS and FP. All authors reviewed the accuracy of the results, discussed the methods, data analysis and presentation of results and reviewed multiple versions of the paper.

## Declaration of Competing Interest

The authors declare that they have no known competing financial interests or personal relationships that could have appeared to influence the work reported in this paper.

## Footnotes

Appendix A Supplementary data (Figs S1 to S8 and Tables A1 to A11) to this article can be found online at https://doi.org/10.1016/j.gloenvcha.2020.102131.
