# Supplementary material for: Multiple cropping systems of the world and the potential for increasing cropping intensity
Source: Glob Environ Change. 2020 Sep;64:102131. doi: 10.1016/j.gloenvcha.2020.102131 (PMC7737095; doi:10.1016/j.gloenvcha.2020.102131)
Supplement: Supplementary data 1 [file mmc1.pdf]

Supplementary Information for

Multiple cropping systems of the world and the potential for increasing cropping intensity

Katharina Waha*, Jan Philipp Dietrich, Felix T. Portmann, Stefan Siebert, Philip K. Thornton, Alberte Bondeau, Mario Herrero

* Correspondence and requests for materials should be addressed to katharina.waha@csiro.au

This PDF file includes:

Figs. S1 to S8

Tables A1 to A11

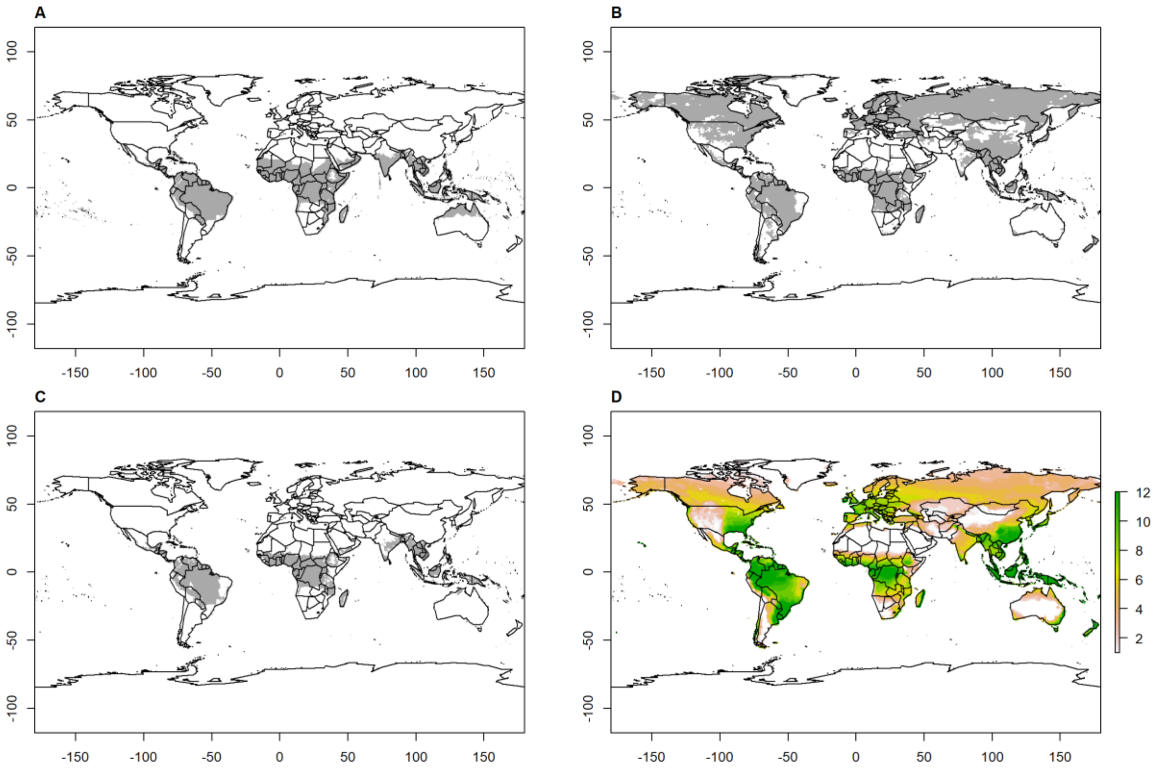

Figure S1. Areas with low frost risk with minimum monthly temperatures higher than or equal to 10°C (a), low drought risk with rainfall variability less than 19% (b) and combined (c). d the potential length of the growing season in months.

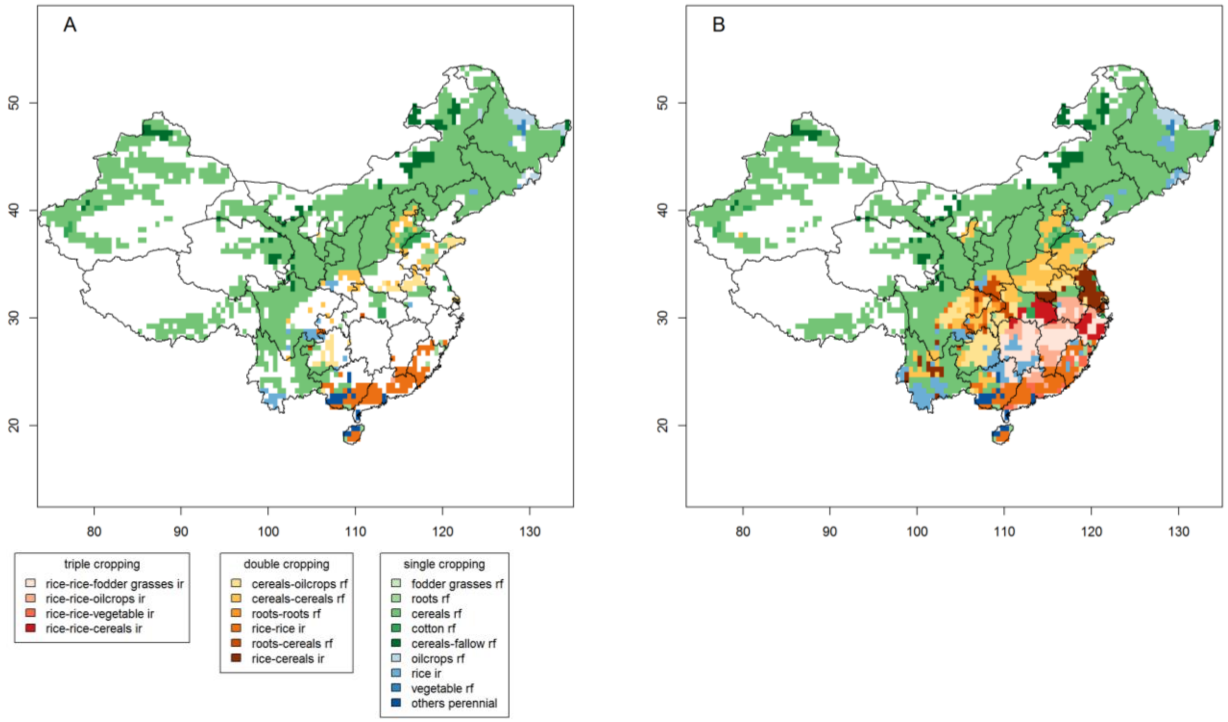

Figure S2. Cropping systems in China. Comparison between global multiple cropping systems dataset as in this study (a) and national data set as in Frolking et al. 2002 (b). Colours represent only one cropping system per 30 arc min grid cell: in a) one that can be found in the global data set and is the system with the largest physical area in Frolking et al. 2002, in b) the system with the largest physical area in Frolking et al. 2002. White areas represent land-use other than cropping or natural vegetation (if found in both maps) or pixels in which the dominant system cannot be found in the global data set (if only in a).

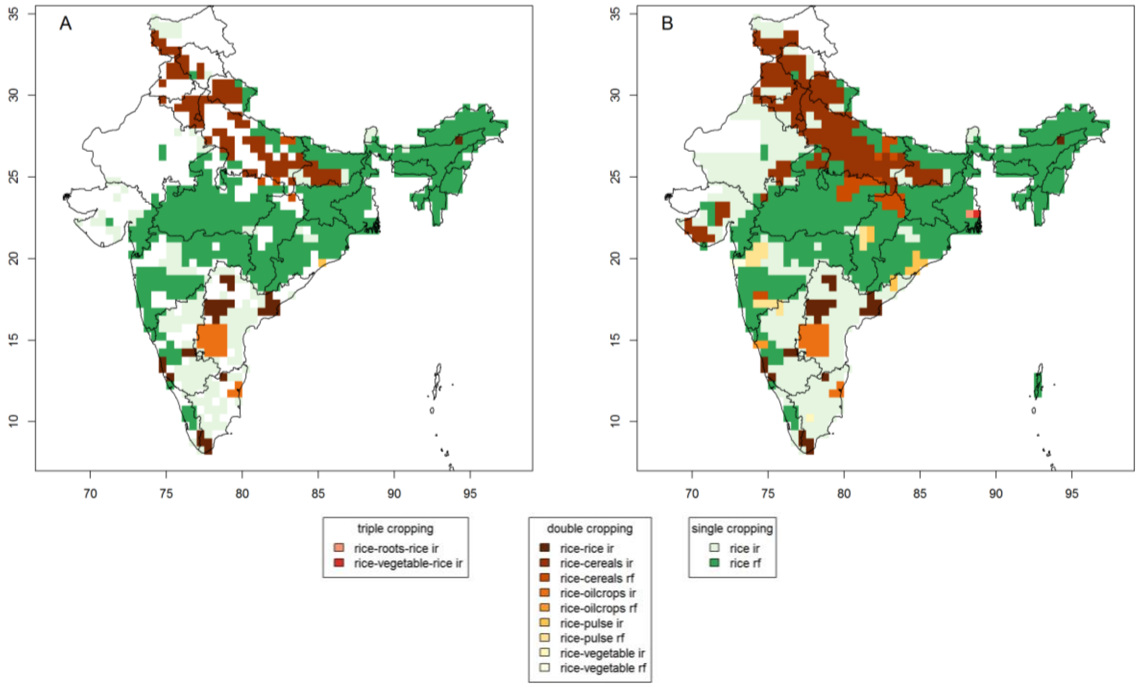

Figure S3. Rice cropping systems in India. Comparison between global dataset as in this study (a) and national data set as in Frolking et al. 2006 (b). Colours represent only one cropping system per 30 arc min grid cell: in a) one that can be found in the global data set and is the system with the largest physical area in Frolking et al. 2006, in b) the system with the largest physical area in Frolking et al. 2006. White areas represent land-use other than cropping or natural vegetation (if found in both maps) or pixels in which the dominant system cannot be found in the global data set (if only in A).

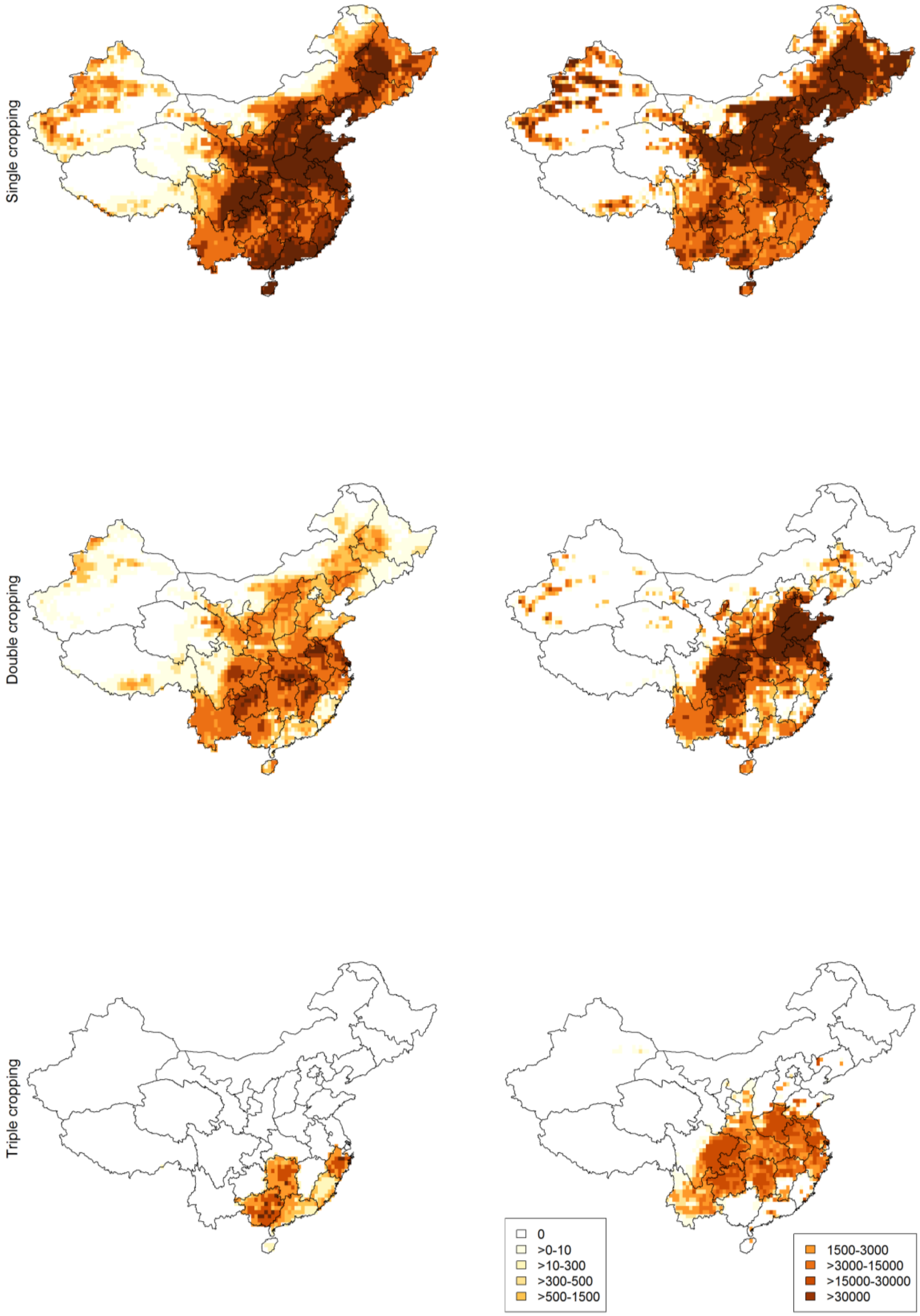

Figure S4. Evaluation of cropping intensity on rainfed cropland in China. Single, double, and triple cropping as in Frolking et al. (2002) (right) and this global classification (left). Cropland is in hectare per 30 arc min grid cell.

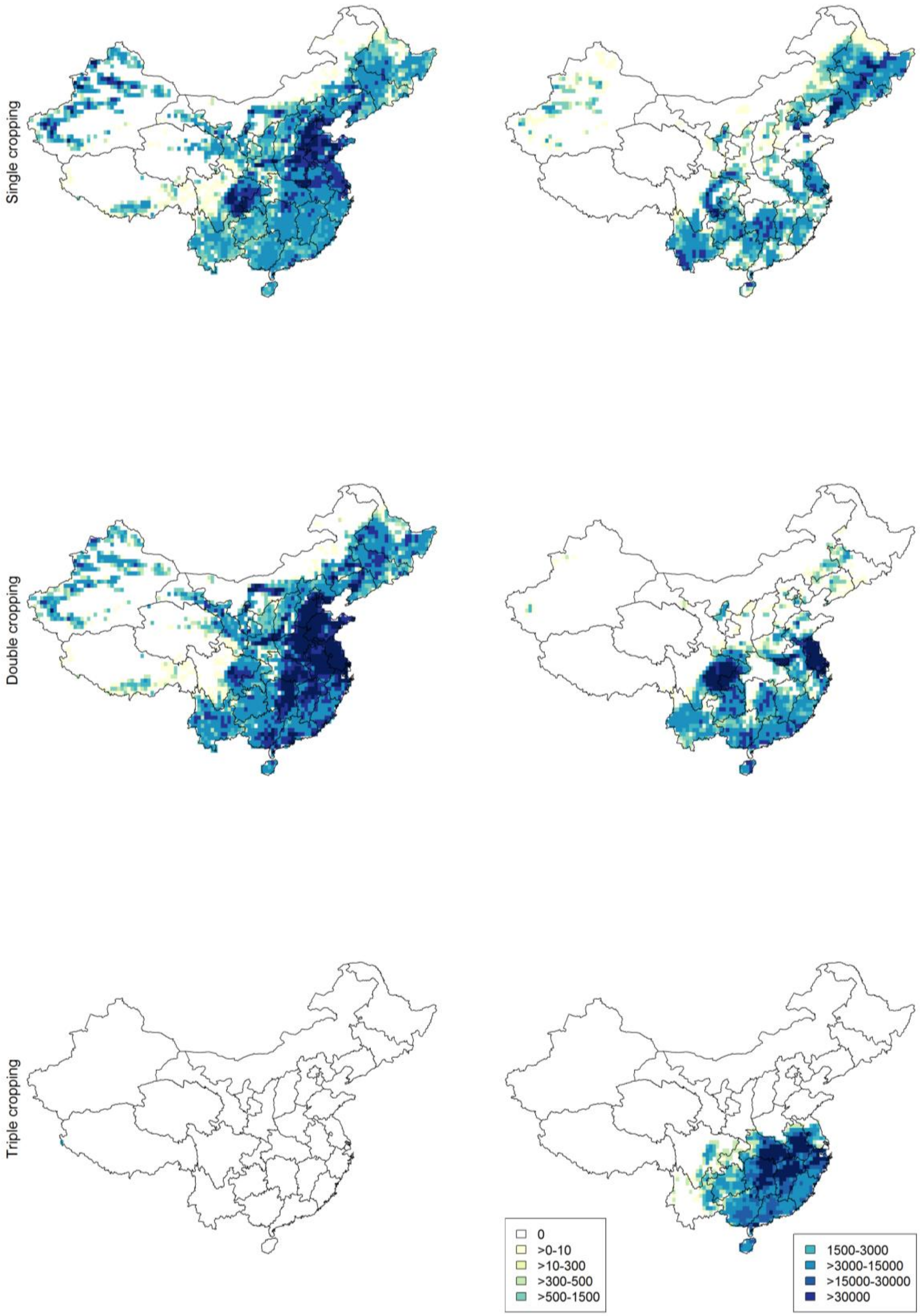

Figure S5. Evaluation of cropping intensity on irrigated cropland in China. Single, double, and triple cropping as in Frolking et al. (2002) (right) and this global classification (left). Cropland is in hectare per 30 arc min grid cell.

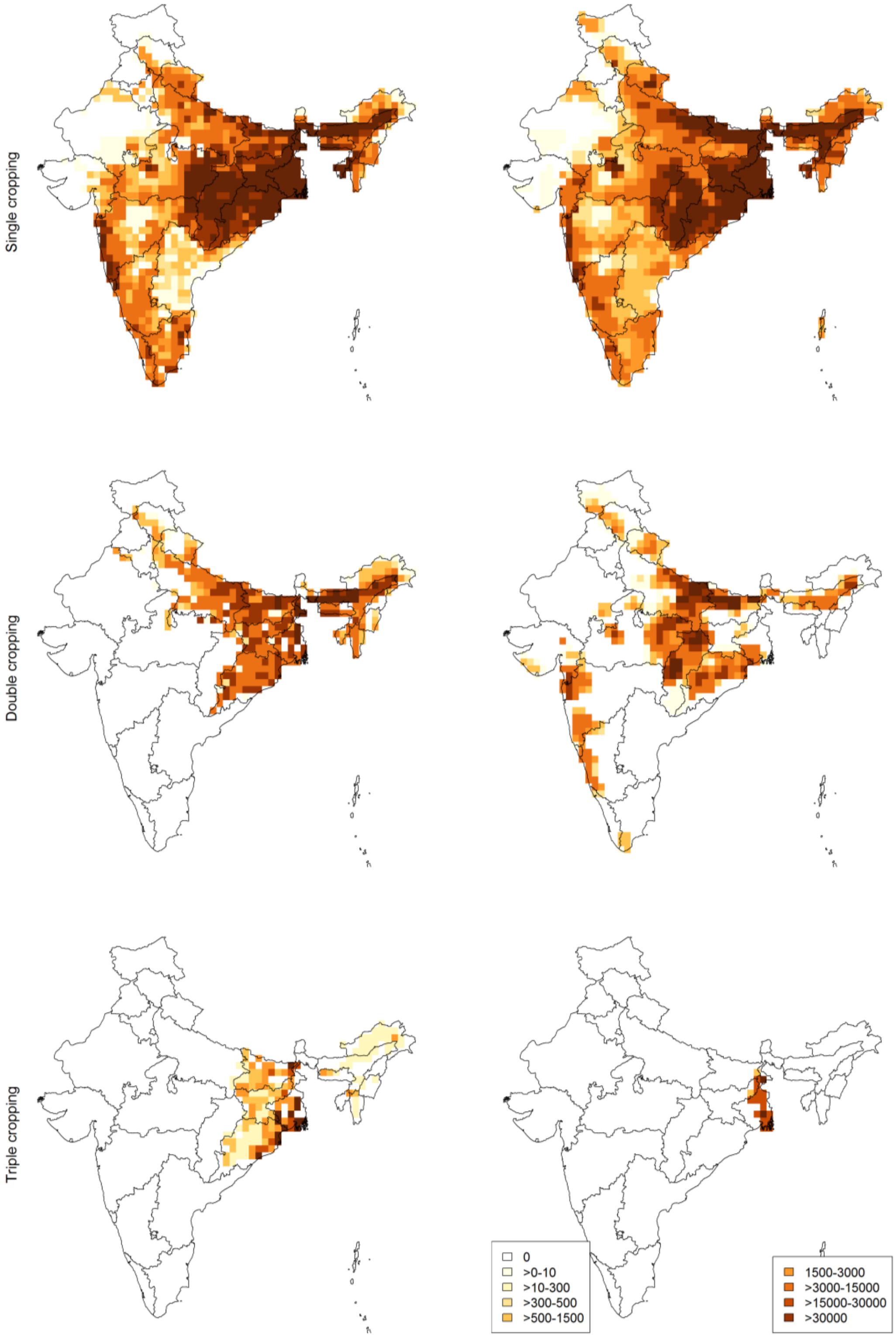

Figure S6. Evaluation of cropping intensity of rainfed cropping systems with <u>rice only</u> in India. Single, double, and triple cropping with rice as in Frolking et al. (2006) (right) and this global classification (left). Cropland is in hectare per 30 arc min grid cell.

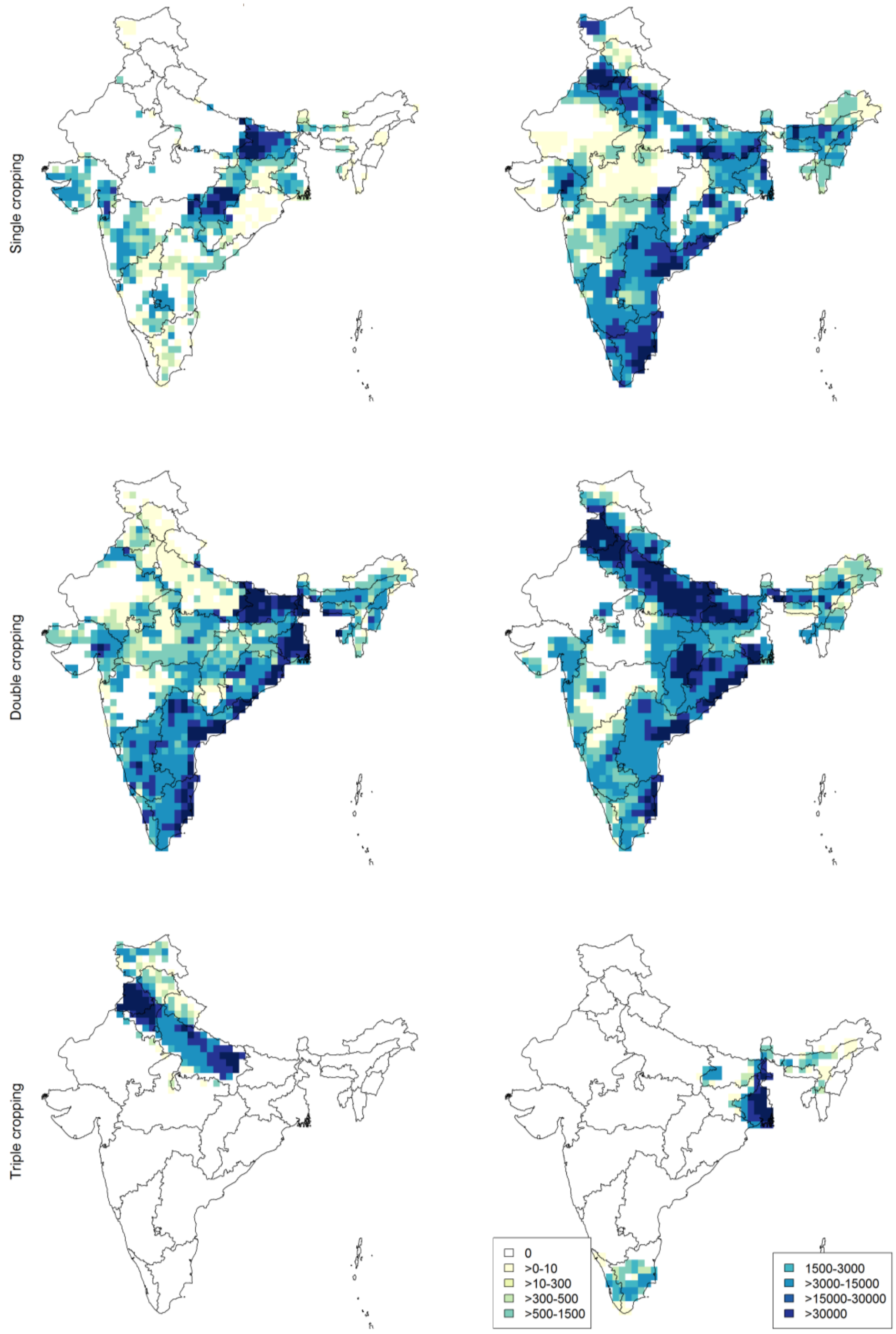

Figure S7. Evaluation of cropping intensity of irrigated cropping systems <u>with rice only</u> in India. Single, double, and triple cropping with rice as in Frolking et al. (2006) (right) and this global classification (left). Cropland is in hectare per 30 arc min grid cell.

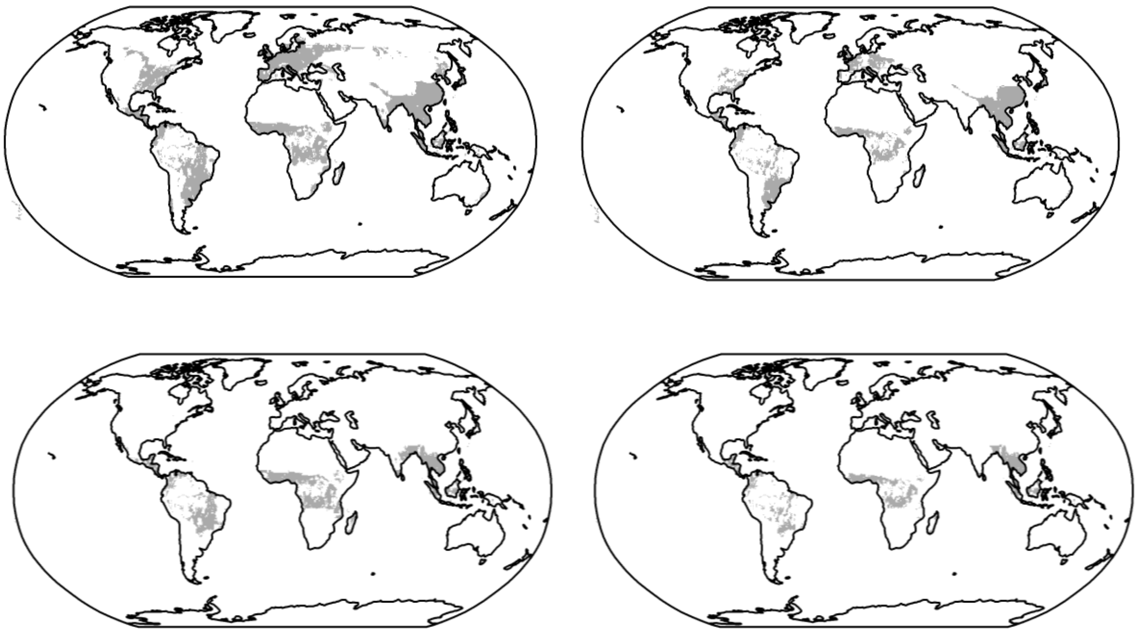

Figure S8. Areas with potential for increasing cropping intensity. Top row: for scenario A2m with at least two months difference between actual and potential growing season (left) and A4m with at least four months difference between actual and potential growing season (right). Bottom row: for scenario A2m- and A4m-, as A2m and A4m but restricted to areas with low frost and drought risk. See materials & methods section in main text for details.

Table A 1 Possible cropping systems as a combination of different crops and sub-crops.

Crop	# sub-crops	Sub-crops indicate	Possible cropping frequencies/systems
**Rice, rainfed**	5	Multiple cropping and different varieties (paddy rice, upland rice, deepwater rice)	Single/Double/Triple cropping with paddy rice (4 mo)<br>Single cropping upland rice (7-8 mo) or deepwater rice (7 mo)
**Rice, irrigated**	2 or 3	Multiple cropping	Double/Triple cropping with paddy rice (4 mo)
**Maize**	2	Multiple cropping and different varieties	Single/Double cropping
**Wheat, Barley (rainfed), Rye**	2	Different varieties (spring and winter)	Double cropping with other crops<br>Single cropping spring wheat (shorter growing period) or winter wheat (longer growing period, planted in August) *
**Others annual, rainfed**	2 or 3	Different varieties	Double/Triple cropping with the same or different crops<br>Single cropping
**Others annual, irrigated**	2, 3, 4 or 5‡	Different varieties	Double/Triple cropping with the same or different crops<br>Single cropping
**Cassava¥**	2	Different varieties (short-growing and long-growing)	Single cropping with a short-growing variety or a long-growing variety<br>Double cropping with a different crop

* Winter and spring wheat or barley are usually not grown consecutively on the same field

‡Others annual irrigated with five sub-crops exists only in Palestine, Jordan and Lebanon

¥ The two cassava varieties never exist in the same administrative unit, therefore formally they are not reported as sub-crops but have distinct growing seasons.

In addition to the crops listed, the crop calendar also reports sorghum double cropping in Yemen (irrigated) and Guatemala (rainfed) and potato double cropping in Morocco (rainfed).

Table A 2 Literature and data sets used to evaluate the global gridded multiple cropping data set.

**Country**	**References**
**China**	(Frolking *et al.*, 2002; Qiu *et al.*, 2003, 2017; Yan *et al.*, 2013; Zuo *et al.*, 2013, 2014; Li *et al.*, 2014)
**South Asia**	(Sheikh, Rehman and Yates, 2003; Frolking, Yeluripati and Douglas, 2006; Gumma *et al.*, 2011, 2016; National Resource Census Project / Indian Space Research Organization, 2012; Yadvinder-Singh *et al.*, 2014; Biradar and Xiao, 2017; Government of India, 2017)
**Africa**	(Mutai and Ward, 2000; Liebmann *et al.*, 2012; Tanzania Ministry of Agriculture, 2012; Kassie *et al.*, 2013; Teklewold *et al.*, 2013; Vrieling, de Leeuw and Said, 2013; Waha *et al.*, 2013; Ouda, Noreldin and Abd El-Latif, 2015; Laborte *et al.*, 2017)
**Latin America**	(Dalrymple, 1971; Limon-Ortega *et al.*, 2006; Calviño and Monzon, 2009; Arvor *et al.*, 2011, 2012; Spera *et al.*, 2014; Andrade and Satorre, 2015; Anderson *et al.*, 2017; Kastens *et al.*, 2017)

Table A 3 Crop area in multiple cropping systems (hectare). Crops not shown are perennial and/or tree crops (oil palm, sugar cane, grapes, coffee, cocoa, citrus, date palm) that do not occur in sequential cropping systems.

Crop	Multiple cropping	Single cropping	Total	% Multiple cropping
**Wheat**	24,122,900	162,967,700	187,090,600	12.9
**Rice**	49,633,400	62,595,600	112,229,000	44.2
**Maize**	13,498,200	124,063,000	137,561,300	9.8
**Millet**	1,747,400	29,988,000	31,735,400	5.5
**Sorghum**	2,260,000	35,178,600	37,438,600	6.0
**Barley**	2,493,700	49,226,000	51,719,700	4.8
**Rye**	230,000	9,851,800	10,081,800	2.3
**Sugar Beet**	125,200	5,914,300	6,039,600	2.1
**Potato**	1,166,200	16,944,000	18,110,200	6.4
**Cassava**	539,300	14,301,800	14,841,200	3.6
**Sunflower**	488,700	19,596,400	20,085,000	2.4
**Soybean**	2,551,900	69,400,500	71,952,400	3.5
**Groundnut**	1,638,600	19,033,000	20,671,500	7.9
**Rapeseed**	5,148,200	14,010,700	19,158,800	26.9
**Cotton**	4,655,300	23,189,800	27,845,200	16.7
**Pulses**	8,226,000	49,930,900	58,156,800	14.1
**Others annual**	15,445,300	75,641,300	91,086,600	17.0
**Total**	133,970,300*	1,020,556,600	1,154,526,900	/

* Difference to the total multiple cropping area presented in Table 1 in main text is due to rounding of numbers to the nearest hundred here.

Table A 4 Total single, cropping and triple cropping areas in China in this study and previous estimates.

	FS02		YH13	QB17	ZL13	This study	
year	1990-1996		2002	2001	2005	1998-2002	
unit	(1000 ha)	(% cropland)	(% cropland)*	(% cropland)	(% cropland)	(1000 ha)	(% cropland)
**Single**	80,158	61.7	66.0	55.0	54.0	77,212	64.1
**Double**	35,741	27.4	32.2	36.4	39.0	42,902	35.6
**Triple**	14,085	10.9	1.8	2.7	7.0	365	0.3
**Total multiple cropping**	49,826	38.3	34.0	39.1	-	43,267	35.9
**Total physical area**	129,984	100	100	100	100	120,480	100
**Total area harvested**	193,895	-	-	-	-	-	-

FS02 is Frolking et al. 2002, QB17 is Qiu et al. 2017, YH13 is Yan et al. 2013, ZL13 is Zuo et al. 2013. *Yan et al. 2013 reported percentage of multiple cropping areas for triple and double cropping. This was converted to percentage of cropland here.

Table A 5 Total single and multiple cropping area of rice and non-rice systems in India in this study and previous estimates.

	BX17	NRSC12	This study	
year	2005	2005/06	1998-2002	
unit	(1000 ha)	(1000 ha)	(1000 ha)	(% cropland)
**Single**	-	82,940¥	125,309	84.0
**Double**	56,000	-	21,462	14.4
**Triple**	1,100	-	2,442	1.6
**Total multiple cropping**	57,800	44,390	23,904	16.0
**Total physical area**	141,160*	127,330	149,214	100
**Total area harvested**	-	-	175,559	-

BX17 is Biradar & Xiao 2017. NRSC12 is National Resource Census Project / Indian Space Research Organization, 2012. * the total cropping area as reported in the census (Government of India, Agricultural Statistics At a Glance 2016, Table 13.2: Selected Categories of Land Use) for comparison purposes. ¥ The single cropping area is the sum of the land use classes 'rabi crop', 'karif crop', 'zaid crop' and 'plantation'

Table A 6 Total single and multiple cropping area of rice and non-rice systems in South Asia in this study and previous estimates. South Asia is the region comprised of India, Bangladesh, Bhutan, Nepal, Pakistan and Sri Lanka.

	**GM16***		**This study**	
year	2010/2011		1998-2002	
unit	(1000 ha)	(% cropland)	(1000 ha)	(% cropland)
**Single**	107,456	51.1	140,441	79.0
**Double**	97,245	46.2	34,781	19.6
**Triple**	5,726	2.7	2,497	1.4
**Total multiple cropping**	102,971	48.9	37,278	21.0
**Total physical area**	210,428	100	177,720	100
**Total area harvested**	319,124	-	217,494	-

GM16 is Gumma et al. 2016. * Sum of full pixel area per respective cropping systems in first column of Table 4

Table A 7 Total single and multiple cropping area of rice systems in India in this study and previous estimates.

	FS06		This study	
year	1999/2000		1998-2002	
unit	(1000 ha)	(% rice area)	(1000 ha)	(% rice area)
**Single**	23,226	57.6	18,762	56.4
**Double**	15,787	39.2	12,037	36.2
**Triple**	1,274	3.2	2,429	7.3
**Total multiple cropping**	17,061	42.3	14,466	43.5
**Total physical area**	40,288	100	33,230	100
**Total area harvested**	58,622	-	50,123	-
FS06 is Frolking et al. 2006				

Table A 8 Total single and multiple cropping area of rice systems in South Asia in this study and previous estimates. South Asia is the region comprised of India, Bangladesh, Bhutan, Nepal, Pakistan and Sri Lanka.

	**GM11***		**This study**	
year	2000/2001		1998-2002	
unit	(1000 ha)	(% rice area)	(1000 ha)	(% rice area)
**Single**	26,486	51.8	22,888	50.4
**Double**	24,638	48.2	20,052	44.1
**Triple**	-	-	2,485	11.0
**Total multiple cropping**	24,638	48.2	22,537	49.6
**Total physical area**	51,124	100	45,425	100
**Total area harvested**	75,762	-	70,477	-

GM11 is Gumma et al. 2011. * Sum of rice 'net' areas for respective cropping intensity from last column in Table 3

Table A 9 Total single and multiple cropping area in selected India states in this study and previous estimates.

	NRSC12				This study			
year	2006/06				1998-2002			
unit	(1000 ha)				(1000 ha)			
state	Uttar Pradesh	Punjab	Bihar	Haryana	Uttar Pradesh	Punjab	Bihar	Haryana
**Single cropping**	5,653	3,210	2,072	1,987	5,401	1,896	2,188	1,347
**Multiple cropping**	9,473	614	3,836	1,065	9,813	2,059	4,055	1,858
**Total physical area**	15,126	3,824	5,908	3,052	15,214	3,955	6,243	3,205

NRSC12 is National Resource Census Project / Indian Space Research Organization, 2012

Table A 10 Potential for increasing cropping intensity on current global croplands by World Bank country groups. Each column shows additional crop area measured as total hectares in a different scenario: A2 and A4, with two or four months difference between potential and actual growing season, respectively and A2- and A4- for cropland with low frost and drought risk Country groups are NAM North America, CAC Central America and Caribbean, ECA Europe and Central Asia, SAM South America, OCE Oceania, MEA Middle East and North Africa, SEA Southeast Asia, SSA Sub-Saharan Africa, SAS South Asia, EAS East Asia.

World region	A2 (ha)	A4 (ha)	A2- (ha)	A4- (ha)
**CAC**	10,427,337	5,790,999	6,215,998	4,877,165
**EAS**	50,338,375	34,512,116	1,316,935	1,282,438
**ECA**	76,385,799	31,060,495	352,787	232,993
**MEA**	3,200,835	892,092	75,887	55,387
**NAM**	62,615,678	12,729,514	75,433	45,588
**OCE**	1,459,886	832,775	41,525	38,704
**SAM**	56,907,783	45,771,704	20,149,555	17,074,350
**SAS**	50,169,031	18,600,466	34,088,758	14,786,887
**SEA**	35,378,623	32,885,364	29,244,501	27,120,192
**SSA**	49,056,298	24,606,543	40,028,788	21,930,102

Table A 11 Potential for increasing cropping intensity on current global croplands by scenario as shown in **Error! Reference source not found.** in the main text. See material and methods in main text for details on scenarios.

	**Difference between actual and potential growing season**					
	< = 2 months			< = 4 months		
	Million hectares	% of single crop area	% of harvested area	Million hectares	% of single crop area	% of harvested area
**All single crop area**	395.6	39.0	30.7	207.3	20.4	16.1
**In areas with T_{min} > 10 °C**	151.7	14.9	11.8	96.3	9.5	7.5
**In areas with CV_{prec} < 19 %**	326.2	32.1	25.3	178.8	17.6	13.9
**In areas with T_{min} > 10 °C and CV_{prec} < 19 %**	131.5	13.0	10.2	87.4	8.6	6.8

T_{min} is the minimum mean monthly temperature for 1970 to 2000 and CV_{prec} is the coefficient of variation of annual rainfall for 1980 to 2000

References

Anderson, W. *et al.* (2017) 'Crop production variability in North and South America forced by life-cycles of the El Nino Southern Oscillation', *Agricultural and Forest Meteorology*. Elsevier B.V., 239, pp. 151–165. doi: 10.1016/j.agrformet.2017.03.008.

Andrade, J. F. and Satorre, E. H. (2015) 'Single and double crop systems in the Argentine Pampas: Environmental determinants of annual grain yield', *Field Crops Research*. Elsevier B.V., 177, pp. 137–147. doi: 10.1016/j.fcr.2015.03.008.

Arvor, D. *et al.* (2011) 'Classification of MODIS EVI time series for crop mapping in the state of Mato Grosso , Brazil', *International Journal of Remote Sensing*, 32(22), pp. 7847–7871. doi: 10.1080/01431161.2010.531783.

Arvor, D. *et al.* (2012) 'Analyzing the agricultural transition in Mato Grosso , Brazil , using satellite-derived indices', *Applied Geography*. Elsevier Ltd, 32(2), pp. 702–713. doi: 10.1016/j.apgeog.2011.08.007.

Biradar, C. M. and Xiao, X. (2017) 'Quantifying the area and spatial distribution of double- and triple-cropping croplands in India with multi-temporal MODIS imagery in 2005', *International Journal of Remote Sensing*, 1161(June). doi: 10.1080/01431160903464179.

Calviño, P. and Monzon, J. (2009) 'Farming Systems of Argentina : Yield Constraints and Risk Management', in Victor Sadras, D. C. (ed.) *Crop Phyiology. Applications for Genetic Improvement and Agronomy*. Elsevier Inc., pp. 55–70. doi: 10.1016/B978-0-12-374431-9.00003-7.

Dalrymple, D. G. (1971) *Survey of multiple cropping in less developed nations*. Washington D.C.: U.S. Department of Agriculture, U.S. Agency for International Development.

Frolking, S. *et al.* (2002) 'Combining remote sensing and ground census data to develop new maps of the distribution of rice agriculture in China', *Global Biogeochemical Cycles*. AGU, 16(4), pp. 10–38. Available at: http://dx.doi.org/10.1029/2001GB001425.

Frolking, S., Yeluripati, J. B. and Douglas, E. (2006) 'New district-level maps of rice cropping in India: A foundation for scientific input into policy assessment', *Field Crops Research*, 98(2–3), pp. 164–177. Available at: http://www.sciencedirect.com/science/article/B6T6M-4JCBKV2-1/2/2a4473dc49a26f9222aff3ac60577906.

Government of India (2017) *Agricultural Statistics at a Glance 2016*. New Delhi, India.

Gumma, M. K. *et al.* (2011) 'Mapping rice areas of South Asia using MODIS multitemporal data Mapping rice areas of South Asia using MODIS multitemporal data', *Journal of Applied Remote Sensing*, 5(1), p. 53547. doi: 10.1117/1.3619838.

Gumma, M. K. *et al.* (2016) 'Mapping rice-fallow cropland areas for short-season grain legumes intensification in South Asia using MODIS 250 m time-series data', *International Journal of Digital Earth*, 9(10), pp. 981–1003. doi: 10.1080/17538947.2016.1168489.

Kassie, B. T. *et al.* (2013) 'Adapting to Climate Variability and Change: Experiences from Cereal-Based Farming in the Central Rift and Kobo Valleys, Ethiopia', *Environmental Management*, 52(5), pp. 1115–1131. doi: 10.1007/s00267-013-0145-2.

Kastens, J. H. *et al.* (2017) 'Soy moratorium impacts on soybean and deforestation dynamics in Mato Grosso, Brazil', *PLoS ONE*, 12(4), pp. 1–21. doi: 10.1371/journal.pone.0176168.

Laborte, A. G. *et al.* (2017) 'RiceAtlas, a spatial database of global rice calendars and production',

Scientific Data, 4, p. 170074. doi: 10.1038/sdata.2017.74.

Li, L. *et al.* (2014) 'Mapping Crop Cycles in China Using MODIS-EVI Time Series', *Remote Sensing*, 6(3), pp. 2473–2493. doi: 10.3390/rs6032473.

Liebmann, B. *et al.* (2012) 'Seasonality of African precipitation from 1996 to 2009', *Journal of Climate*, 25(12), pp. 4304–4322. doi: 10.1175/JCLI-D-11-00157.1.

Limon-Ortega, A. *et al.* (2006) 'Soil aggregate and microbial biomass in a permanent bed wheat – maize planting system after 12 years', *Field Crops Research*, 97, pp. 302–309. doi: 10.1016/j.fcr.2005.11.001.

Mutai, C. C. and Ward, M. . (2000) 'East African Rainfall and the Tropical Circulation / Convection on Intraseasonal to Interannual Timescales', *Journal of Climate*, 13, pp. 3915–3939.

National Resource Census Project / Indian Space Research Organization (2012) *Natural Resource Census - Land Use Land Cover Database, Version 1.0*. Available at: bhuvan.nrsc.gov.in/gis/thematic (Accessed: 10 July 2018).

Ouda, S., Noreldin, T. and Abd El-Latif, K. (2015) 'Water requirements for wheat and maize under climate change in North Nile Delta', *Spanish Journal of Agricultural Research*, 13(1), p. e03–001 (10). doi: doi.org/10.5424/ sjar/2015131-6412.

Qiu, B. *et al.* (2017) 'Mapping cropping intensity trends in China during 1982–2013', *Applied Geography*. Elsevier Ltd, 79, pp. 212–222. doi: 10.1016/j.apgeog.2017.01.001.

Qiu, J. *et al.* (2003) 'Mapping Single-, Double-, and Triple-crop Agriculture in China at 0.5° × 0.5° by Combining County-scale Census Data with a Remote Sensing-derived Land Cover Map', *Geocarto International*, 18(2), pp. 3–13. doi: 10.1080/10106040308542268.

Sheikh, A. D., Rehman, T. and Yates, C. M. (2003) 'Logit models for identifying the factors that influence the uptake of new "no-tillage" technologies by farmers in the rice–wheat and the cotton–wheat farming systems of Pakistan's Punjab', *Agricultural Systems*, 75(1), pp. 79–95.

Spera, S. A. *et al.* (2014) 'Recent cropping frequency, expansion, and abandonment in Mato Grosso, Brazil had selective land characteristics', *Environmental Research Letters*, 9(6), p. 64010. Available at: http://stacks.iop.org/1748-9326/9/i=6/a=064010.

Tanzania Ministry of Agriculture, F. S. and C. (2012) *National sample census of Agriculture 2007/2008. Smallholder agriculture. Volume II : Crop Sector - National report*. Dar es Salaam.

Teklewold, H. *et al.* (2013) 'Cropping system diversification , conservation tillage and modern seed adoption in Ethiopia: Impacts on household income, agrochemical use and demand for labor', *Ecological Economics*. Elsevier B.V., 93, pp. 85–93. doi: 10.1016/j.ecolecon.2013.05.002.

Vrieling, A., de Leeuw, J. and Said, M. Y. (2013) 'Length of Growing Period over Africa: Variability and Trends from 30 Years of NDVI Time Series', *Remote Sensing*, 5, pp. 982–1000.

Waha, K. *et al.* (2013) 'Adaptation to climate change through the choice of cropping system and sowing date in sub-Saharan Africa', *Global Environmental Change*, 23(1), pp. 130–143. doi: 10.1016/j.gloenvcha.2012.11.001.

Yadvinder-Singh *et al.* (2014) 'Improving Water Productivity of Wheat-Based Cropping Systems in South Asia for Sustained Productivity', in *Advances in Agronomy*. Elsevier, pp. 157–258. doi: 10.1016/B978-0-12-800131-8.00004-2.

Yan, H. *et al.* (2013) ‘Multiple cropping intensity in China derived from agro-meteorological observations and MODIS data’, *Chinese Geographical Science*, 24(2), pp. 205–219. doi: 10.1007/s11769-013-0637-2.

Zuo, L. *et al.* (2014) ‘Developing grain production policy in terms of multiple cropping systems in China’, *Land Use Policy*. Elsevier Ltd, 40, pp. 140–146. doi: 10.1016/j.landusepol.2013.09.014.

Zuo, L. J. *et al.* (2013) ‘Spatial exploration of multiple cropping efficiency in china based on time series remote sensing data and econometric model’, *Journal of Integrative Agriculture*. Chinese Academy of Agricultural Sciences, 12(5), pp. 903–913. doi: 10.1016/S2095-3119(13)60308-1.
